# Identification of *Salvia miltiorrhiza* germplasm resources based on metabolomics and DNA barcoding

**DOI:** 10.3389/fphar.2024.1518906

**Published:** 2025-01-07

**Authors:** Gaojie He, Jinhui Man, Ying Chen, Xiaoqin Zhang, Xin Wang, Kelu An, Laha Amu, Wenqin Chen, Baowei Wang, Yue Shi, Xiaohui Wang, Shengli Wei

**Affiliations:** ^1^ School of Chinese Materia Medica, Beijing University of Chinese Medicine, Beijing, China; ^2^ Engineering Research Center of Good Agricultural Practice for Chinese Crude Drugs, Ministry of Education, Beijing, China; ^3^ School of Biology and Medicine, Beijing City University, Beijing, China; ^4^ Modern Research Center for Traditional Chinese Medicine, Beijing Institute of Traditional Chinese Medicine, Beijing University of Chinese Medicine, Beijing, China

**Keywords:** *Salvia miltiorrhiza*, targeted metabolomics, chloroplast genome, DNA barcoding, germplasm resource identification

## Abstract

**Introduction:**

*Salvia miltiorrhiza* radix et rhizoma (Danshen) is a crucial medicinal material for treating cardiovascular and cerebrovascular diseases. However, the presence of adulterants and intraspecific variability poses challenges to its clinical safety.

**Methods:**

This study collected samples of *S. miltiorrhiza* from various regions and commonly encountered adulterants. The composition differences of *S. miltiorrhiza* radix and its adulterants were analyzed by fingerprint and broad-target metabolomics. Chloroplast genome was used to distinguish intra-genus species and DNA barcoding was used to identify germplasm sources.

**Results:**

The fingerprinting analysis proved that there is no chemical composition consistency between *S. miltiorrhiza* radix and its adulterants. Broad-targeted metabolomics can distinguish *S. miltiorrhiza* radix from *Salvia yunnanensis* radix, *Dipsacus asperoides* radix, and *Arctium lappa* radix. Additionally, comparative chloroplast genome analysis indicated that *atpF* and *rps4-trnT-UGU* were the potential DNA barcodes for *S. miltiorrhiza*. 259 samples from 13 provinces and 21 origins were amplified and sequenced, resulting in the identification of 62 haplotypes. The unique haplotypes found in Shanxi Luoyang, Shandong Qingdao and other places can be used as molecular geographic markers for the identification of the germplasm source of *S. miltiorrhiza*.

**Discussion:**

This study systematically differentiates *S. miltiorrhiza* from its adulterants and highlights the potential of unique haplotypes as markers for sourcing. The findings provide strong scientific evidence for the clinical safety of *S. miltiorrhiza*, emphasizing the importance of proper cultivation, selection, and breeding of varieties.

## 1 Introduction

Danshen, the dried roots and rhizomes of *Salvia miltiorrhiza*. (NationalPharmacopoeiaCommittee, 2020), is widely used in China, the United States and Japan (Cheng, 2006; Pang et al., 2016). It was firstly characterized in *Shennong Bencao Jing* (200–300 AD, Han Dynasty). The traditional clinical uses of *S. miltiorrhiza* radix include activating blood circulation and removing blood stasis, nourishing blood and tranquilizing the mind, and cooling blood and dispersing carbuncles. Available studies have shown that diterpenes and phenolic acids have a variety of pharmacological activities, the main components of which include tanshinone ⅡA, cryptotanshinone, salvianolic acid A-E, tanshinol, etc., (Lam et al., 2006; Zhou et al., 2011; Mei et al., 2019). Salvianolic acid from *S. miltiorrhiza* radix has anti-inflammatory and antioxidant activities, cardioprotective effects, and can be clinically used for the treatment of thrombosis and hypertension (Ho and Hong, 2011; Zhang et al., 2016). Tanshinone has powerful anticancer effects both *in vivo* and *in vitro*, and is also used in the treatment and prevention of atherosclerosis and coronary heart disease (Liu et al., 2016; Zhao D. et al., 2016). In addition, *S. miltiorrhiza* radix is used in the treatment of Alzheimer’s disease as well as neurodegenerative insomnia (Fang et al., 2010; Li et al., 2015).

*S. miltiorrhiza* radix is widely used in clinics because of its rich pharmacological effects and good pharmacodynamic effects. With the increasing clinical usage of *S. miltiorrhiza* radix, the cultivation area of *S. miltiorrhiza* has been expanded and some problems have been highlighted. Studies have shown that the content of active ingredients in *S. miltiorrhiza* radix is affected by a variety of factors (Su et al., 2015). In the market, some herbs imitate *S. miltiorrhiza* radix, such as *Dipsacus asperoides* radix and *Arctium lappa* radix (Ma et al., 2012; Zhu et al., 2019). Moreover, due to the relatively similar morphology of plants within the *Salvia* genus, they are also easily confused with *S. trijuga* radix, *S. przewalskii* radix, S. *digitaloide* radix, *S. yunnanensis* radix*,* and so on (Pang et al., 2016; Liang et al., 2019), which is not conducive to the safety of clinical use of the drug. *S. miltiorrhiza* has a wide geographic distribution and a complex environment, resulting in complex intraspecies resources, and the morphology of *S. miltiorrhiza* plants originating from different regions varies markedly, with variations in traits such as leaf morphology, flower color, and root morphology (Li et al., 2009; Peng et al., 2014). Therefore, *S. miltiorrhiza* is involved in the complexity of resources among other plant species and within *Salvia* species, and the efficient identification of *S. miltiorrhiza* interspecies and intraspecies is of great significance for standardization of *S. miltiorrhiza* cultivation, clinical application, resource protection, and origin tracing, and is the main direction of the present study to be tackled.

The common identification methods for traditional Chinese medicine (TCM) include morphological, microscopic, physicochemical, chemical fingerprinting, DNA barcoding, chloroplast genomics, and metabolomics methods. The chemical fingerprint analysis of traditional Chinese medicine was proposed by the State Food and Drug Administration (SFDA) in 2000 and has been widely used for the authentication, origin identification, and quality evaluation of Chinese herbal medicines and related preparations. By comparing *S. miltiorrhiza* radix from different origins and its counterfeit *S. przewalskii* radix through hierarchical cluster analysis, significant differences could be detected between them. They further selected salvianolic acid B, rosmarinic acid, cryptotanshinone, tanshinone I, and tanshinone IIA as evaluation standards (Zhong G. X. et al., 2009). The fingerprints of 13 batches of *S. miltiorrhiza* radix were also identified by UPLC combined with chemometric analysis (Luo et al., 2015).

The concept of metabolomics was first introduced by Fiehn et al. (2000). Plant metabolomics has now been widely applied in the field of TCM, including the identification of medicinal materials, quality evaluation, changes before and after processing of medicinal materials, and the effects under stress conditions (Ye et al., 2016; Awin et al., 2019; Mun et al., 2020; Wang C. X. et al., 2021). For example, using untargeted metabolomics, 11 identifying markers were found in 85 samples of *Coptidis* rhizoma from three different origins, samples from different origins could be successfully differentiated with 100% accuracy (Cao et al., 2021). Similarly, the differential compounds between Chuanwu and processed Chuanwu could be successfully identified by metabolomics methods (Li et al., 2013).

With the development of plant identification research, DNA barcoding technology has emerged, and many scholars have used this technique for molecular-level identification of plants in nature (Wang et al., 2023). Currently, chloroplast genomes have been applied to various aspects such as plant evolutionary analysis, species origin identification, molecular marker development, and genetic diversity analysis, achieving significant scientific results (Liu et al., 2018; Shidhi et al., 2021; Guo et al., 2022). Chloroplast DNA fragments primarily fall into two categories: one includes universal barcodes such as *trnH-psbA*, *matK*, and *rbcL*; the other consists of specifically selected barcodes. Single fragments may not accurately identify all medicinal plants, but multi-gene combinations provide more variable site information, offering higher accuracy in species identification. Moon et al. distinguished *Fritillariae Thunbergii* and *Fritillariae Hupehensis Bulbus* and five *Fritillaria* species using the *matK* and *rps16* genes (Moon et al., 2018).

Each technique has its unique advantages and limitations. The combined application of multiple techniques can not only complement each other but also validate the results obtained. For example, the combination of metabolomics based on ultra-high-performance liquid chromatography-quadrupole time-of-flight mass spectrometry (UHPLC-QTOF-MS) and DNA barcoding methods based on *trnH-psbA* and *ITS2* barcodes has been used to distinguish between *S. yangii* and *S. abrotanoides* (Bielecka et al., 2021).

Although some scholars have distinguished *S. miltiorrhiza* radix and its adulterants (Cao et al., 2021) or *S. miltiorrhiza* from different regions (Zhong G. X. et al., 2009), the number of samples and geographic scope of the identifications were limited, and only a rough differentiation between *S. miltiorrhiza* radix and its adulterants or some of the production regions was made. This study employed a combination of methods to systematically distinguish *S. miltiorrhiza* radix and its adulterants, as well as different origins of *S. miltiorrhiza*. Specifically, *S. miltiorrhiza* radix and its adulterants could be distinguished by chemical fingerprints. Three chemical markers were selected by broad-targeted metabolomics to differentiate *S. miltiorrhiza* radix from different origins. *S. miltiorrhiza* from its intraspecific species could be differentiated by chloroplast genomes. Finally, by comparing the chloroplast genomes of different origins, specific barcodes *atpF* and *rps4-trnT-UGU* were identified, which can be used to identify the germplasm origin of *S. miltiorrhiza* from different origins.

## 2 Materials and methods

### 2.1 Plant materials

The plant samples were collected from different production area by ourselves in 2021–2022, and were identified by Prof. Shengli Wei of the School of Chinese Materia Medica, Beijing University of Chinese Medicine. Among them, the samples used for HPLC fingerprint experiment contain 10 batches of *S. miltiorrhiza* radix samples from different producing areas, 6 batches of *S. yunnanensis* radix, 6 batches of *S. przewalskii* radix, 6 batches of *D. asperoides* radix, and 5 batches of *A. lappa* radix (Supplementary Table S1). The samples used for the metabolomics experiment include the roots and rhizomes of ten *S. miltiorrhiza* and three *S. yunnanensis*, as well as the roots of three *D. asperoides* and three *A. lappa* (Supplementary Table S2). The fresh plant of *S. miltiorrhiza* samples (Sm01∼Sm09) were collected from different production area and used for the chloroplast genome analysis (Supplementary Table S3). The fresh young leaves collected were quickly dehydrated and dried in silica gel for chloroplast genome DNA extraction. A total of 259 *S. miltiorrhiza* samples were collected from 21 different production area in 13 provinces and were used for DNA barcoding analysis (Supplementary Table S4).

### 2.2 HPLC-UV analysis

*S. miltiorrhiza* radix and its adulterants were crushed to prepare sample solution, and appropriate amount of salvianolic acid B, cryptotanshinone, tanshinone Ⅰ and tanshinone ⅡA were weighed to prepare standard solution. The chromatograph was Shimadzu LC-40D high performance liquid chromatograph. The chromatographic column was Waters XBridge®C18 column (250 mm × 4.6 mm, 5 um) with mobile phase consisting of acetonitrile (A) and 0.1% phosphoric acid water (B). The elution procedure was referred to Zhu et al. (2023): 0 min 80% B, 15 min 74% B, 22 min 60% B, 29 min 53% B, 49 min 37% B, 57 min 31% B, 72 min 10% B, 75 min 10% B. The flow rate was 1 mL·min^−1^. The detection wavelength was set to 275 nm. The injection volume was 10 μL and the column temperature was set at 20°C.

### 2.3 Widely targeted metabolomics analysis

The samples of *S. miltiorrhiza* radix and its adulterants were crushed and extracted for HPLC-MS experiment. The solution to be measured was obtained through 0.22 μm filtration membrane. The mass spectrometer used was the QTRAP 6500 Plus. The parameters were set as follows: ion source temperature was 500°C, spray voltage (IS) was 5500 V (positive mode)/−5500 V (negative mode). Ion source gas I (GS1), gas II (GS2), and curtain gas (CUR) were set at 40, 40, and 25 psi, respectively.

Qualitative analysis of compounds was performed based on the self-built database WT-PMDB from Huada BGI. Skyline software was used for relative quantitative analysis of the data. Analyst 1.6.3 software was used to process mass spectrometry data. MetaboAnalyst (https://www.metaboanalyst.ca/MetaboAnalyst/) was used for principal component analysis (PCA) and partial least squares discriminant analysis (PLS-DA) of the samples to observe sample grouping.

### 2.4 Chloroplast genome analysis

Total DNA from *S. miltiorrhiza* leaves was extracted using the E. Z.N.A Plant DNA kit (D3485, OMEGA, United States). The quality of the extracted DNA was evaluated by 1.0% agarose gel electrophoresis, and the DNA concentration and purity were determined using a NanoDrop micro-spectrophotometer (Thermo Scientific, United States). Sequencing was performed using the Illumina sequencing platform.

The annotated chloroplast genome of *S. miltiorrhiza* (GenBank accession number: JX312195.1) available in the NCBI database was used as a reference. The raw sequences obtained after sequencing were quality checked using FastQC, and Trimmomatic software was used to filter out adapter sequences and low-quality regions. NOVOPlasty was used to assemble the raw reads into complete chloroplast genomes. BWA was used to align high-quality reads back to the chloroplast genome sequence, and manual inspection was performed in IGV to ensure accurate assembly. The complete chloroplast genome sequence was annotated using CPGAVAS software, and tRNA genes were identified and manually corrected using tRNAscan-SE software. The chloroplast genome map was drawn online using Organellar Genome DRAW (http://ogdraw.mpimp-golm.mpg.de/cgi-bin/ogdraw.pl).

Long repeat sequences in the chloroplast genome were analyzed using the online software REPuter (http://bibiserv.techfak.uni-bielefeld.de/reputer/) with parameters set to Hamming distance of 3, maximum computed repeats of 5,000, and minimal repeat size of 30. Simple sequence repeat (SSR) sequences in the chloroplast genome were analyzed using the online software MISA (http://pgrc.ipk-gatersleben.de/misa/) with threshold parameters set to 1, 2, 3, 4, 5, and 6, and nucleotide parameters set to 10, 5, 4, 3, 3, and 3, respectively. The minimum distance between two SSRs was set to 100 bp. Tandem repeat sequences were detected using the Tandem Repeats Finder software (https://tandem.bu.edu/trf/trf.html) with default settings.

A phylogenetic tree was constructed using 24 chloroplast genome sequences from 22 species of the *Salvia* genus published on NCBI (Supplementary Table S5) and the nine chloroplast genome sequences of *S. miltiorrhiza* obtained in this study. The chloroplast genome sequences of 23 species were aligned using the online software MAFFT 7 (https://mafft.cbrc.jp/alignment/server/), and the aligned sequences were imported into MEGA X software. The neighbor-joining (NJ) method was used to construct the phylogenetic tree, with bootstrap set to 1,000.

### 2.5 Identification of specific *S. miltiorrhiza* DNA barcodes

The chloroplast genome of *S. miltiorrhiza* was aligned using the mVISTA online tool (http://genome.lbl.gov/vista/index.shtml) with the shuffle-LAGAN mode. Multi-sequence alignment analysis of gene regions and intergenic regions of the nine chloroplast genomes of *S. miltiorrhiza* was performed using ClustalX software to observe the number of different chloroplast genomes that can be distinguished by different sequences and their similarity.

Based on the selected sequences, two fragment-specific primers were designed using Primer 5 software (Supplementary Table S6) for amplification of samples from different origins of *S. miltiorrhiza*. The total PCR reaction volume was 50 μL, including 5 µL of 10 × Taq buffer, 4 µL of dNTP Mix (2.5 mmol·L^−1^), 1 μL of 0.1% BSA, 1 µL of Taq DNA polymerase (2.5 U·µL^-1^), 2 µL of 10 µmol primer, and ddH_2_O up to 50 µL.

The PCR amplification procedures included predenaturation at 94°C for 5 min, 94°C denaturation for 30, renaturation for 30 s (*atpF* annealing temperature of 51.3°C and *rps4-trnT-UGU* annealing temperature of 54.2°C), and 72°C for 1 min (35 cycles); a 72°C extension for 10 min; and storage at 4°C.

The amplified PCR products were analyzed by 1% agarose gel electrophoresis. The amplification results were observed under UV light. The gel containing the target DNA bands was excised quickly. The gel-purified PCR products were recovered and purified using the Vazyme PCR product recovery kit. The purified products were sent to Beijing Liuhe Huada Genomics Institute for bidirectional sequencing.

The sequences were manually checked and edited using Chromas software, assembled using Contig Express, and low-quality sequences were removed. The assembled sequences were imported into DNAMAN 9.0 for alignment analysis. DnaSP software was used to calculate the number of segregating sites (S), the number of haplotypes (h), haplotype diversity (h_d_), and nucleotide diversity (Pi) for *S. miltiorrhiza* populations. ArcMap was employed to construct haplotype geographic distribution maps. MEGAX was used to calculate genetic distances for combined analysis fragments, and a neighbor-joining method was applied to construct haplotype phylogenetic trees.

## 3 Results

### 3.1 HPLC fingerprint analysis could identify *S. miltiorrhiza* radix and the adulterants

**FIGURE 1:**
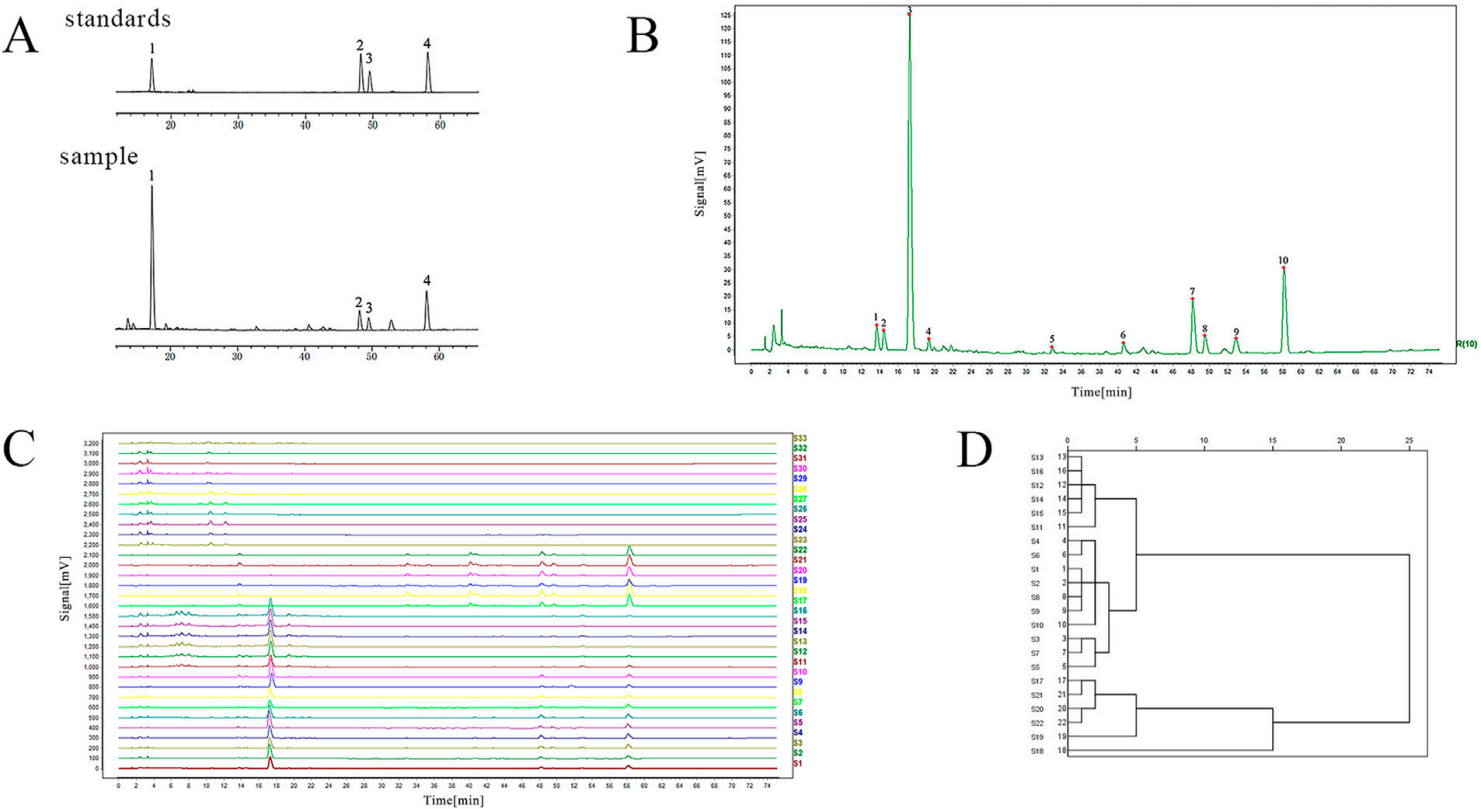
The HPLC fingerprint of *S. miltiorrhiza* radix and its adulterants. **(A)** The peak charts of the standard and sample solutions (1: salvianolic acid B; 2: cryptotanshinone; 3: tanshinone I; 4: tanshinone IIA). **(B)** The chromatogram of ten common peaks of *S. miltiorrhiza* radix. **(C)** HPLC fingerprints of *S. miltiorrhiza* radix and its adulterants (*S. miltiorrhiza* radix: S1∼S10; *S. yunnanensis* radix: S11∼S16; *S. przewalskii* radix: S17∼S22; *D. asperoides* radix: S23∼S28; *A. lappa* radix: S29∼S33). **(D)** Systematic clustering dendrogram of four components of *S. miltiorrhiza* radix.

To identify *S. miltiorrhiza* radix and the adulterants, we first performed HPLC fingerprint analysis. The separation degree of the four components specified in the 2020 edition of the Chinese Pharmacopoeia had met the requirements (Figure 1A). The chromatographic peaks of the four standard components contained in the sample solution were identified, where peak 1 corresponded to salvianolic acid B, peak 2 corresponded to cryptotanshinone, peak 3 corresponded to tanshinone I, and peak 4 corresponded to tanshinone IIA. The chromatographic fingerprints of 10 batches of *S. miltiorrhiza* radix from different origins were imported into the Chinese Medicine Chromatographic Fingerprint Similarity Evaluation System (2012 Edition) software. Using the chromatographic profile of S3 *S. miltiorrhiza* radix as the reference fingerprint, multi-point correction was applied to match the chromatographic profiles of the 10 batches of *S. miltiorrhiza* radix. Finally, 10 common peaks were determined, and a reference chromatogram was generated using the median method (Figure 1B). Among them, peak 3 corresponded to salvianolic acid B, peak 7 corresponded to cryptotanshinone, peak 8 corresponded to tanshinone I, and peak 10 corresponded to tanshinone IIA. The methodological investigation experiments showed that the instrument precision, method reproducibility and sample stability were good.

All obtained batch samples were imported into the Traditional Chinese Medicine Chromatographic Fingerprint Similarity Evaluation System workstation to obtain a comprehensive fingerprint map containing 10 batches of *S. miltiorrhiza* radix, 6 batches each of *S. yunnanensis* radix, *S. przewalskii* radix, *D. asperoides* radix, and 5 batches of *A. lappa* radix (Figure 1C). The fingerprint spectra of multiple batches showed significant differences between *S. miltiorrhiza* radix (S1-S10) and *D. asperoides* radix (S23-S28) and *A. lappa* radix (S29-S33), indicating that *S. miltiorrhiza* radix has many different chemical components from the latter two. However, the peak shapes of *S. miltiorrhiza* radix (S1-S10) were more similar to those of *S. yunnanensis* radix (S11-S16) and *S. przewalskii* radix (S17-S22), suggesting that they share more similar chemical components. From 4 to 10 min, *S. yunnanensis* radix exhibited more chromatographic peaks than *S. miltiorrhiza* radix. From 46 to 60 min, the overall peak area of *S. yunnanensis* radix was lower than that of *S. miltiorrhiza* radix, indicating that *S. yunnanensis* radix contains more water-soluble components, but *S. miltiorrhiza* radix contains richer tanshinones compounds. Compared to *S. przewalskii* radix, *S. miltiorrhiza* radix showed more chemical components with larger peak areas from 12 to 24 min. From 30 to 60 min, *S. przewalskii* radix had more components eluted, and the overall peak area of the three tanshinones components (tanshinone IIA, cryptotanshinone, and tanshinone I) was larger than that of *S. miltiorrhiza* radix, indicating that *S. przewalskii* radix contains more lipophilic components and fewer water-soluble components than *S. miltiorrhiza* radix.

The intra-group similarity of fingerprint profiles was calculated for *S. miltiorrhiza* radix, *S. yunnanensis* radix, *S. przewalskii* radix, *D. asperoides* radix and *A. lappa* radix, respectively. Using S3 as the standard reference chromatogram for *S. miltiorrhiza* radix, the intra-group similarity of 10 batches of *S. miltiorrhiza* radix is shown in Supplementary Table S7. The similarity between each batch and the reference chromatogram was greater than 0.9700, indicating good intra-group similarity. The similarity among samples from different regions of *S. miltiorrhiza* radix was greater than 0.9300, indicating good consistency within the group. Using S11 as the standard reference chromatogram for *S. yunnanensis* radix, the intra-group similarity of *S. yunnanensis* radix was analyzed (Supplementary Table S8). All samples showed high similarity with the reference chromatogram, with a similarity greater than 0.9900, and the similarity among different samples was also higher than 0.9900, indicating good consistency within the group. Using S17 as the standard reference chromatogram for *S. przewalskii* radix, the intra-group similarity of *S. przewalskii* radix was analyzed (Supplementary Table S9). The similarity of all samples with the reference chromatogram was greater than or equal to 0.9900, and the similarity among different samples was greater than 0.9700, indicating good consistency within the group. Using S23 as the standard reference chromatogram for *D. asperoides* radix, the intra-group similarity of *D. asperoides* radix was analyzed (Supplementary Table S10). The similarity of all samples with the reference chromatogram was greater than 0.9200, and the similarity among different samples was also greater than 0.8000, indicating good consistency within the group. Using S29 as the standard reference chromatogram for *A. lappa* radix, the intra-group similarity of *A. lappa* radix was analyzed (Supplementary Table S11). The similarity of all samples with the reference chromatogram was not less than 0.8900, and except for the similarity between S33 and S31, which was 0.7820, the similarity among different batches was greater than 0.8300, indicating good consistency within the group.

The content determination indicators specified in the 2020 edition of the Chinese Pharmacopoeia, including salvianolic acid B, cryptotanshinone, tanshinone I, and tanshinone IIA, were selected as analytical variables. System clustering was performed based on the peak areas of these four components in *S. miltiorrhiza* radix, *S. yunnanensis* radix, and *S. przewalskii* radix. The results show that the clustering can be broadly categorized into three groups: *S. miltiorrhiza* radix, *S. yunnanensis* radix, and *S. przewalskii* radix (Figure 1D). This suggests that there is no consistency among these three herbs in terms of the four selected chemical components used for systematic clustering.

### 3.2 Metabolomic could distinguish different origins of *S. miltiorrhiza* radix and the adulterants

**FIGURE 2:**
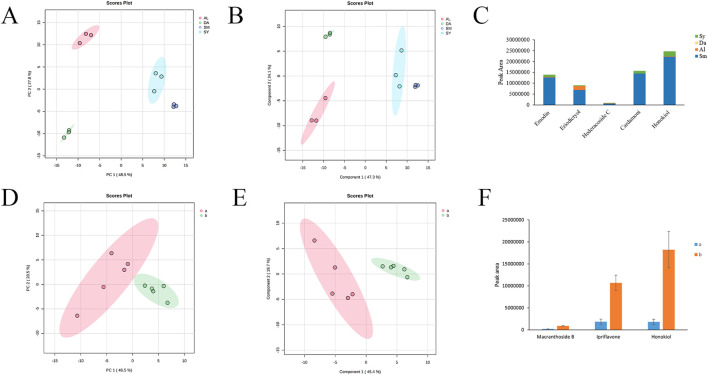
Broad-Targeted metabolomic analysis of *S. miltiorrhiza* radix and its adulterants. **(A)** PCA scores of *S. miltiorrhiza* radix, *D. asperoides* radix, *A. lappa* radix and *S. yunnanensis* radix (SM: *S. miltiorrhiza*; AL: *A. lappa* radix; DA: *D. asperoides* radix; SY: *S. yunnanensis* radix). **(B)** PLS-DA scores of *S. miltiorrhiza* radix, *D. asperoides* radix, *A. lappa* radix and *S. yunnanensis* radix (SM: *S. miltiorrhiza* radix; AL: *A. lappa* radix; DA: *D. asperoides* radix; SY: *S. yunnanensis* radix). **(C)** Peak areas of differential metabolites of *S. miltiorrhiza* radix and its adulterants (Sm: *S. miltiorrhiza* radix; Al: *A. lappa* radix; Da: *D. asperoides* radix; Sy: *S. yunnanensis* radix). **(D)** PCA scores of *S. miltiorrhiza* radix from different origins (a: Sichuan, Shanxi, Hebei, Henan, Shaanxi; b: Anhui, Jiangxi, Liaoning, Shandong, Gansu). **(E)** PLS-DA scores of *S. miltiorrhiza* radix from different origins (a: Sichuan, Shanxi, Hebei, Henan, Shaanxi; b: Anhui, Jiangxi, Liaoning, Shandong, Gansu). **(F)** Relative peak area share of different compounds of *S. miltiorrhiza* radix from different origins (a: Sichuan, Shanxi, Hebei, Henan, Shaanxi; b: Anhui, Jiangxi, Liaoning, Shandong, Gansu).

Based on ten samples of *S. miltiorrhiza* radix from different origins and three samples each of *S. yunnanensis* radix, *D. asperoides* radix and *A. lappa* radix, a broad-targeted metabolomic analysis was conducted to detect and analyze metabolites. A total of 387 compounds were detected, including flavonoids, amino acids and derivatives, organic acids, terpenes, and other primary and secondary metabolites (Supplementary Table S12). The PCA score plot shows that the data from different species fall within their respective confidence intervals (Figure 2A). The model had an R^2^ value of 0.9910 and a p value of 0.0010, indicating a good model with reliable predictions. The species are clearly separated from each other and relatively clustered within their own groups, indicating different metabolic patterns among *S. miltiorrhiza* radix, *D. asperoides* radix, *A. lappa* radix, and *S. yunnanensis* radix. To further elucidate these differences, a supervised analysis method, PLS-DA, was used to analyze the data of the four species. The model had an R^2^ value of 0.8970 and a Q^2^ value of 0.6700, indicating a good model with reliable predictions. The results show that *S. miltiorrhiza* radix, *D. asperoides* radix, *A. lappa* radix, and *S. yunnanensis* radix can be clearly distinguished (Figure 2B). Using fold change values and variable importance in the projection (VIP) from the PLS-DA model as selection criteria (fold change >2, VIP >1.5) (Supplementary Tables S13, S14), differential metabolites between samples were screened. Finally, five differential compounds were selected, namely Hederacoside C, Cardamoni, Emodin, Eriodictyol, and Honokiol (Figure 2C). These five differential compounds are predominantly present in *S. miltiorrhiza* radix and can be used to differentiate it from *D. asperoides* radix, *A. lappa* radix, and *S. yunnanensis* radix.

The broad-targeted metabolomic data of *S. miltiorrhiza* radix from ten different origins were subjected to PCA analysis. The model had an R^2^ value of 0.5560 and a p value of 0.0090. As shown in Figure 2D, the samples from ten production areas of *S. miltiorrhiza* radix were divided into two main clusters. Cluster a includes samples from Sichuan, Shanxi, Hebei, Henan, and Shaanxi, while Cluster b includes samples from Anhui, Jiangxi, Liaoning, Shandong, and Gansu. This suggests that samples from similar geographical regions of *S. miltiorrhiza* radix share similar chemical compositions. PLS-DA analysis was performed on the samples from ten production areas of *S. miltiorrhiza* radix. The model had an R^2^ value of 0.9970 and a Q^2^ value of 0.8710. The significant differences between the two groups of samples can be observed from Figure 2E, suggesting that there may be distinct differences in metabolites between *S. miltiorrhiza* radix from Sichuan, Shanxi, Hebei, Henan, and Shaanxi, and those from Anhui, Jiangxi, Liaoning, Shandong, and Gansu. Using fold change values and VIP from the PLS-DA model as selection criteria (fold change, VIP >2) (Supplementary Tables S15, S16), three differential metabolites were selected between samples. These metabolites are Ipriflavone, Macranthoside B, and Honokiol. Overall, the content of these three differential compounds is higher in samples from Anhui, Jiangxi, Liaoning, Shandong, and Gansu production areas (Figure 2F). These three compounds can serve as biomarkers for distinguishing *S. miltiorrhiza* radix from different production areas.

### 3.3 Chloroplast genome and phylogenetic tree for identification within the genus

**FIGURE 3:**
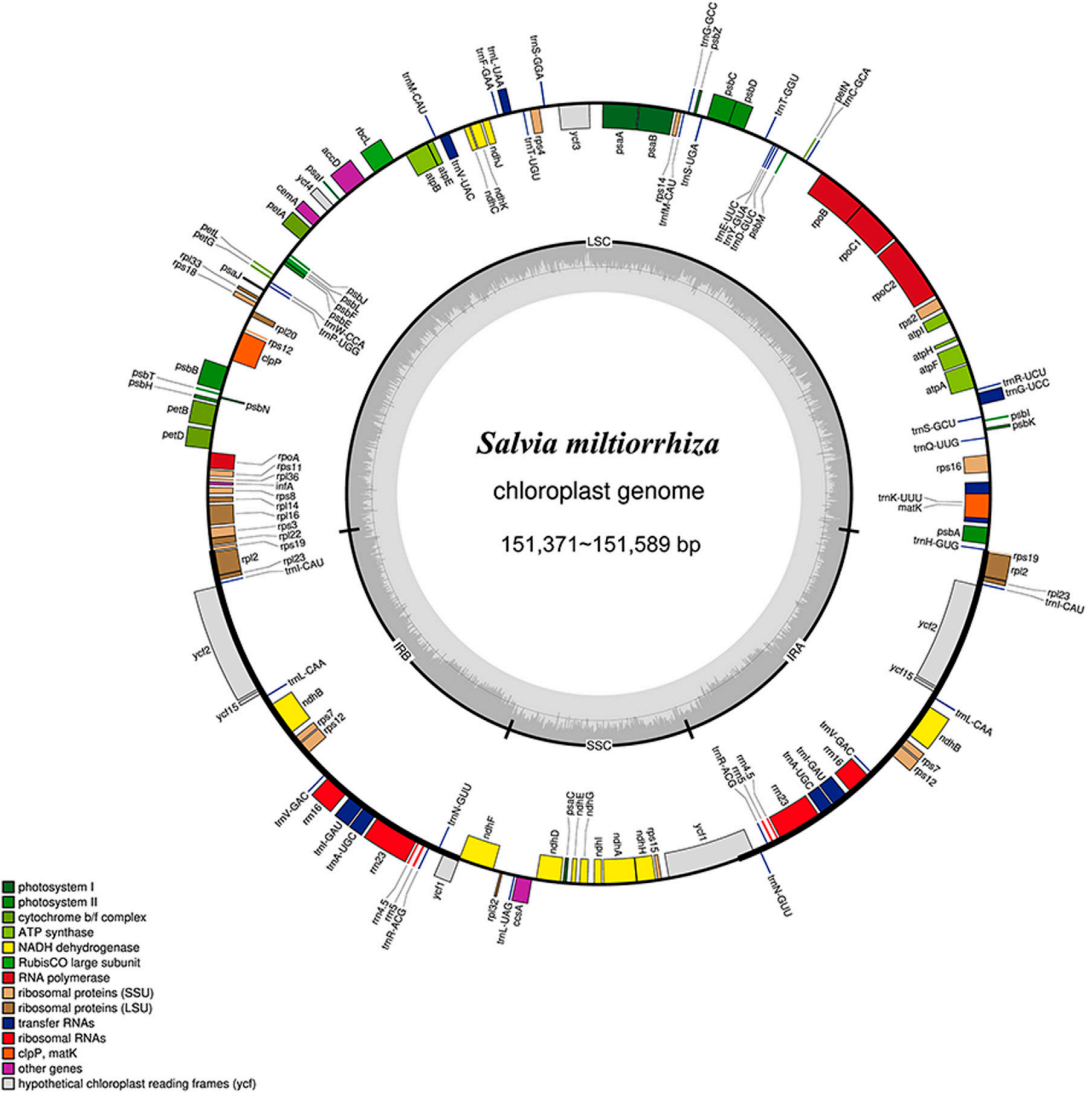
Chloroplast genome map of *S. miltiorrhiza*. Thick lines indicate the extent of the inverted repeat regions (Ira and Irb), which separate the genome into small (SSC) and large (LSC) single copy regions. Genes drawn inside the circle are transcribed clockwise, and those outside are transcribed counterclockwise. Different colors represent different functional groups of genes.

The chloroplast genomes of *S. miltiorrhiza* from different origins were assembled, annotated, and analyzed. The results (Supplementary Table S17; Figure 3) showed that the total length of the chloroplast genome sequences from nine different origins of *S. miltiorrhiza* ranged from 151,371 to 151,589 bp, which is similar to other angiosperms and exhibits a typical circular quadripartite structure. The length of the large single copy region (LSC) ranged from 82,753 to 82,841 bp (36.10%–36.14%), the small single copy region (SSC) ranged from 17,572 to 17,591 bp (31.98%–32.05%), and the inverted repeat regions (IRs) ranged from 25,521 to 25,590 bp (43.10%–43.14%). These findings provide insights into the chloroplast genome features of *S. miltiorrhiza* and lay the foundation for further phylogenetic analyses.

**FIGURE 4:**
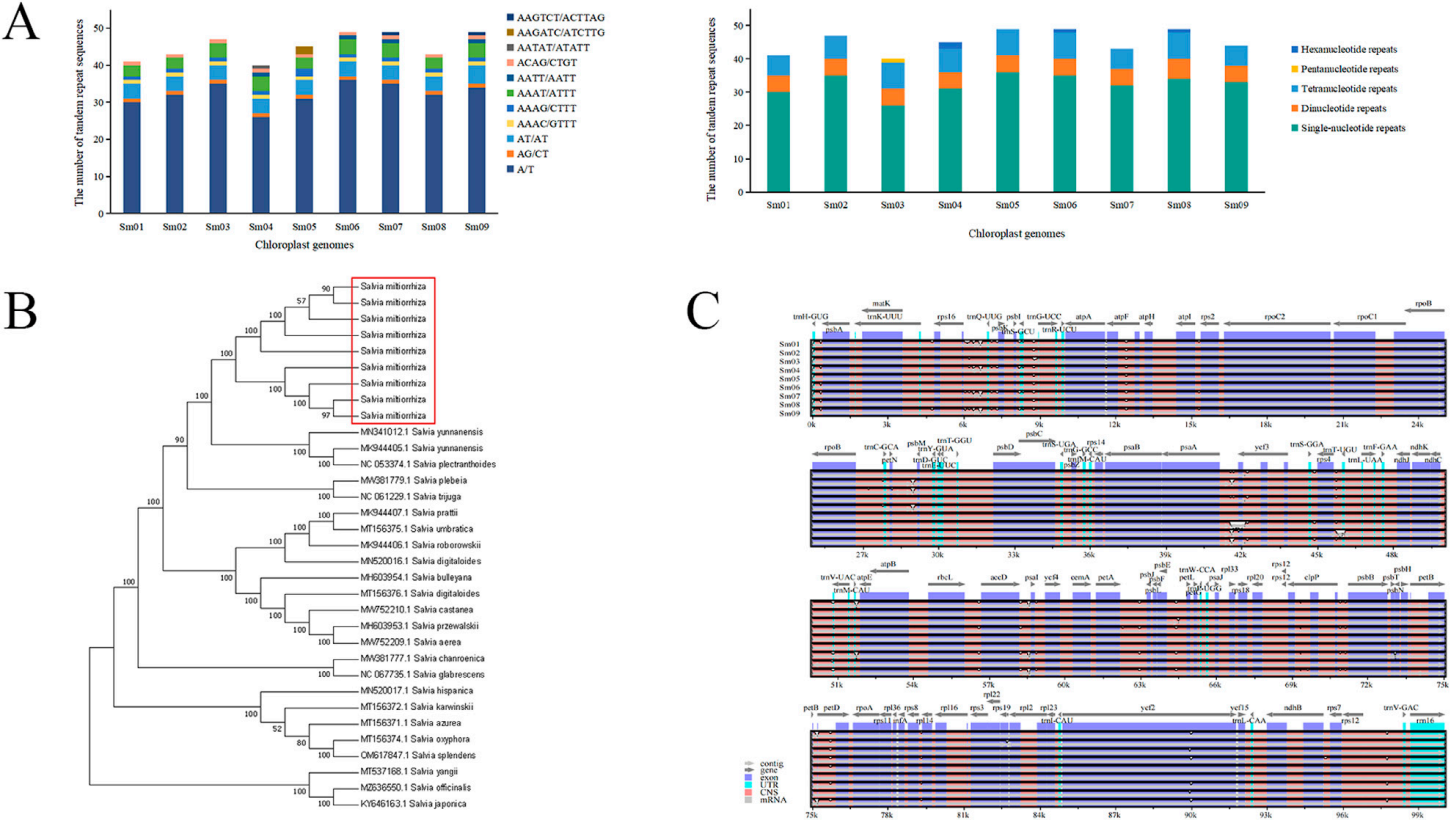
*S. miltiorrhiza* chloroplast genomes analysis. **(A)** The number of different types of simple repeat sequences in different chloroplast genomes (left) and the number of different repeat types in different chloroplast genomes (right). **(B)** Phylogenetic tree of 33 chloroplast genomes in the *Salvia* species. **(C)** Intraspecific global comparison of nine *S. miltiorrhiza* chloroplast genomes.

The chloroplast genomes of *S. miltiorrhiza* from nine different origins have consistent gene numbers (Supplementary Table S18), each containing 132 genes (including 18 repeated genes), consisting of 87 protein-coding genes (CDS), 37 tRNA genes, and 8 rRNA genes. These genes can be classified into 4 major categories and 18 subcategories based on their functions: genes related to photosynthesis and self-replication are relatively abundant, while other genes and those with unknown functions are generally less represented. Among these genes, 18 different genes contain introns, with 15 genes containing a single intron and 3 protein-coding genes containing 2 introns (*ycf3, clpP, rps12*). The genes *ndhB, rpl2, rpl23, rps12, rps7, rrn16, rrn23, rrn4.5, rrn5, trnA-UGC, trnI-CAU, trnI-GAU, trnL-CAA, trnN-GUU, trnR-ACG, trnV-GAC, ycf15,* and *ycf2* are located in the inverted repeat regions, with each gene having a duplicate. The results of REPuter long repeat sequence analysis showed that the nine *S. miltiorrhiza* chloroplast genomes had 43∼55 long repeat sequences, and forward and palindromic repeats were the more abundant types, with 21∼26 forward repeats, 22∼26 palindromic repeats, and 0 reverse and complementary repeats (Supplementary Table S19). Most of the repeats were in the range of 30∼39 bp in length, followed by 40∼49 bp. The number of tandem repeat sequences in nine *S. miltiorrhiza* chloroplast genomes was 14∼23. Simple repeat sequence analysis of different *S. miltiorrhiza* chloroplast genomes by MISA showed that there were 40∼49 SSRs in nine *S. miltiorrhiza* chloroplast genomes, and a total of 407 SSRs were identified, which were mainly single-nucleotide repeats with the repeat type of A/T, followed by tetranucleotide repeats with the repeat types of AAAC/GTTT, AAAG/CTTT, AAAT/ATTT, AATT/AATT, ACAG/CTGT, followed by dinucleotide repeats as AG/CT, AT/AT repeats, and a smaller number of pentanucleotide AATAT/ATATATT and hexanucleotide AAGATC/ATCTTG, AAGTCT/ACTTAG repeats, with no trinucleotide repeats identified (Figure 4A).

24 annotated chloroplast genomes of *Salvia* species from NCBI, along with the 9 chloroplast genomes of *S. miltiorrhiza* sequenced in this study, were used to construct a NJ phylogenetic tree. The results (Figure 4B) showed that the 9 chloroplast genomes of *S. miltiorrhiza* formed a single branch, with 100% support, clustering with *S*. *yunnanensis* and *S*. *plectranthoides*, indicating a close relationship between *S. miltiorrhiza* and the two species. The phylogenetic tree revealed that chloroplast genomes could be used for identification of *S. miltiorrhiza* and other species within the *Salvia* genus, achieving intragenus discrimination of *S. miltiorrhiza*.

### 3.4 Identification of *S. miltiorrhiza* germplasm resources based on specific DNA barcodes

Using the annotated chloroplast genome of *S. miltiorrhiza* as a reference, nine chloroplast genomes from different origins of *S. miltiorrhiza* were compared intraspecifically using the mVISTA online software (Figure 4C). The results showed that variation within *S. miltiorrhiza* was relatively conservative, with larger variations observed in intergenic regions compared to coding regions. Regions such as *rps16-trnQ-UUG, atpF, ycf3, rps4-trnT-UGU, accD-psaI, petB-petD,* and *clpP* exhibited greater variability compared to other genes or intergenic regions. Analysis of the nine chloroplast genomes of *S. miltiorrhiza* using ClustalX software revealed that the sequences of *ndhF, atpF, ycf3, clpP, rpl32, rps7-ndhB, atpA-atpF, trnS-GCU-trnG-UCC, rrn4.5-rrn5, trnG-GCC-trnfM-CAU, petB-petD, psbI-trnS-GCU, rps16-trnQ-UUG, rps8-to-rpl14,* and *rps4-trnT-UGU* could distinguish the nine chloroplast genomes with low sequence similarity (Supplementary Table S20). Combining the analysis results from mVISTA, *rps16-trnQ-UUG, atpF, ycf3, rps4-trnT-UGU, petB-petD,* and *clpP* could be preliminary candidates for DNA barcoding of *S. miltiorrhiza*.

Based on the previous data analysis results, fragments with a length range of 300–1,300 bp, containing a high number of variable sites, and with relatively high amplification efficiency were further selected. The *atpF* and *rps4-trnT-UGU* fragments were ultimately chosen as the research fragments, and specific primers were designed. DNA extraction, PCR amplification, product purification, and sequence comparison were conducted for 259 samples from 21 different production areas in 13 provinces and cities. The experiment showed that the two selected specific fragments could efficiently amplify 259 samples of *S. miltiorrhiza* from different production areas, with amplification efficiencies of 98.0% (*atpF*) and 92.7% (*rps4-trnT-UGU*), respectively. Both fragments successfully amplified a total of 235 samples (Supplementary Figure S1).

After alignment, the length of the *atpF* sequence was determined to be 977 bp, producing a total of 35 variable sites upon sequencing. There were 23 insertion/deletion sites (251∼257 bp, 339∼344 bp, 441 bp, 404∼408 bp, 536∼538 bp, 605 bp), accounting for 65.7% of the variations. There were 12 nucleotide substitutions (56 bp, 280 bp, 308 bp, 405 bp, 411 bp, 455 bp, 517 bp, 687 bp, 745 bp, 809 bp, 901 bp, 929 bp), accounting for 34.3% (Supplementary Table S21). Thus, a total of 34 haplotypes were formed, named aHap1∼aHap34. The length of the *rps4-trnT-UGU* sequence after alignment was 407 bp, and analysis revealed 57 variable sites upon sequencing. Among them, there were 50 insertion/deletion sites (53∼57 bp, 101∼107 bp, 150∼167 bp, 221∼240 bp), accounting for 87.7% of the variations. There were 7 nucleotide substitutions (80 bp, 184 bp, 213 bp, 245 bp, 250 bp, 277 bp, 286 bp), accounting for 12.3% (Supplementary Table S22). Thus, 22 haplotypes were formed, named rHap1∼rHap22.

**FIGURE 5:**
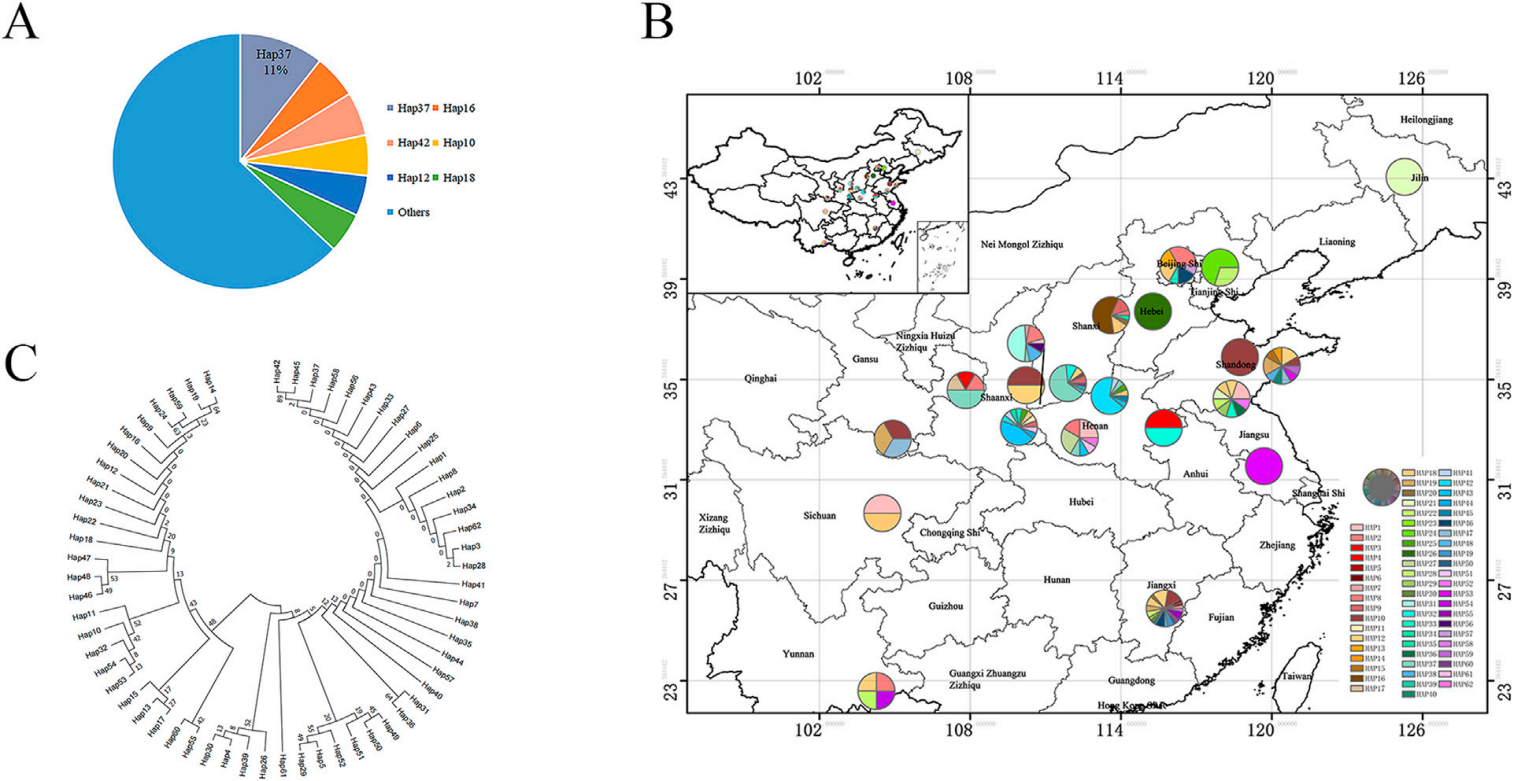
*S. miltiorrhiza* haplotype analysis. **(A)** Percentage of joint haplotypes. **(B)** Geographic distribution of *S. miltiorrhiza* joint sequence frequencies. **(C)** Phylogenetic tree of 62 joint haplotypes of *S. miltiorrhiza*.

The length variation in both *atpF* and *rps4-trnT-UGU* sequences was mainly caused by insertion and deletion of bases. Upon combined analysis of the two fragments, a total of 62 haplotypes were formed among the 235 samples, named Hap1∼Hap62. Among them, Hap37 was the predominant haplotype, found in 25 samples distributed in Shanxi Yuncheng, Shanxi Linfen, Henan Nanyang, Henan Luoyang, and Gansu Qingyang, accounting for 11%. Haplotypes with frequencies higher than 5% included Hap16, Hap42, Hap10, Hap12, and Hap18, while the rest were relatively less abundant (Figure 5A).

The distribution of these 62 haplotypes in different regions was statistically analyzed (Supplementary Table S23). Results revealed that AHBZ (Anhui Bozhou), HBBD (Hebei Baoding), HNLY (Henan Luoyang), and YNWS (Yunnan Wenshan) each possessed a unique haplotype, namely Hap3, Hap26, Hap45, and Hap54, respectively. SDQD (Shandong Qingdao), GSQY (Gansu Qingyang), BJHR (Beijing Huairou), BJMY (Beijing Miyun), and SXLF (Shanxi Linfen) each had two unique haplotypes. HBSJZ (Hebei Shijiazhuang), SDLY (Shandong Linyi), and SXYC (Shanxi Yuncheng) had three unique haplotypes each. HNNY (Henan Nanyang) and SXSL (Shaanxi Shangluo) had four unique haplotypes each. JXGZ (Jiangxi Ganzhou) had 10 unique haplotypes. In total, 19 unique haplotypes were distributed among the populations, indicating rich haplotype diversity. These region-specific haplotypes can be used as DNA barcode markers for regional identification, facilitating the identification of geographical origins.

### 3.5 *S. miltiorrhiza* has rich genetic diversity and small genetic differences

To create the geographic distribution frequency map of the 62 haplotypes using ArcMap, locations like Changchun in Jilin, Weifang in Shandong, Baoding in Hebei, and Yangzhou in Jiangsu each have one haplotype, indicating relatively pure haplotypes (Figure 5B). There are three populations with 10 or more haplotypes: Qingdao in Shandong with 10 haplotypes, Shangluo in Shaanxi with 11 haplotypes, and Ganzhou in Jiangxi with 19 haplotypes, indicating high genetic diversity in these areas. Among the 62 haplotypes, there are 21 shared haplotypes. The most widely distributed shared haplotype is Hap18, found in seven different locations including Shijiazhuang in Hebei, Deyang in Sichuan, Ganzhou in Jiangxi, Shangluo in Shaanxi, Linyi in Shandong, Huairou in Beijing, and Luoyang in Henan. The next most widespread haplotype is Hap10, found in six locations including Qingdao in Shandong, Linfen in Shanxi, Ganzhou in Jiangxi, Weifang in Shandong, Weinan in Shaanxi, and Longnan in Gansu.

Using DnaSP software, genetic diversity parameters of the chloroplast gene combined analysis fragment of *S. miltiorrhiza* were calculated (Supplementary Table S24). The range of haplotype diversity (h_d_) among the 21 populations of *S. miltiorrhiza* varied from 0 to 1, nucleotide diversity (Pi) ranged from 0 to 4.5200, the number of segregating sites (S) ranged from 0 to 11, and the number of haplotypes (h) ranged from 1 to 10. These results indicate that different geographical regions of *S. miltiorrhiza* exhibit differences in genetic diversity. The population JXGZ (Jiangxi Ganzhou) harbored the richest variation in haplotype number and segregating sites, with 11 and 10, respectively. The population BJMY (Beijing Miyun) had the highest h_d_ and Pi values, reaching 1 and 4.52, respectively. Overall, the 21 populations exhibited relatively high genetic diversity, with 71.43% of populations having h_d_ > 0.5.

Genetic distances were calculated for the 62 haplotypes obtained from the combined analysis. The average genetic distance among the 62 haplotypes was 0.0023, ranging from 0.0000 to 0.0054. Hap42 and Hap45 exhibited the largest genetic distance of 0.00536 with Hap24, Hap59, Hap10, Hap32, Hap53, and Hap54. Overall, the genetic distances among the haplotypes were small, indicating minor genetic differences between them. A phylogenetic tree of the 62 combined haplotypes of *S. miltiorrhiza* was constructed using MEGAX software (Figure 5C). It can be observed that the 62 haplotypes have close genetic relationships with each other, with small genetic distances between them. The predominant haplotype, Hap37, is closely related to Hap42 and Hap45.

## 4 Discussion

The correct origin of traditional Chinese medicine (TCM) germplasm is a crucial guarantee for the safety and effectiveness of clinical medication. Due to the adulteration by unscrupulous merchants and the similarity of *S. miltiorrhiza* to other species of the same genus, the germplasm resources of *S. miltiorrhiza* have become confused, affecting clinical efficacy (Li et al., 2009; Zhu et al., 2019). Therefore, it is essential to study the quality consistency of *S. miltiorrhiza* and its adulterants, as well as the germplasm identification of *S. miltiorrhiza*.

The chemical fingerprint of TCM is a comprehensive and quantifiable spectrum obtained from the determination of the complex chemical components in TCM. It is a commonly used method for evaluating the consistency of TCM quality (Zhong X. K. et al., 2009; Liang et al., 2010). In this study, the fingerprints of *S. miltiorrhiza* radix and its adulterants *S. yunnanensis* radix, *S. przewalskii* radix, *D. asperoides* radix and the *A. lappa* radix were determined. The results showed that significant differences in peak shapes between *S. miltiorrhiza* radix and *D. asperoides* radix, and the *A. lappa* radix, suggesting many different chemical components between *S. miltiorrhiza* radix and these two, indicating no quality consistency and allowing differentiation through chemical fingerprints (Figure1C). The spectra of *S. miltiorrhiza* radix, *S. yunnanensis* radix, and *S. przewalskii* radix were more similar, indicating many similar chemical components, making differentiation more challenging. However, our study found that compared to *S. miltiorrhiza* radix, the content of tanshinones was lower in *S. yunnanensis* radix, while *S. przewalskii* radix had a higher content of tanshinone components. Other studies in recent years have reached similar conclusions. The fingerprints of lipophilic component fingerprints of 16 batches of *S. miltiorrhiza* radix and 18 batches of *S. yunnanensis* radix were compared and found to be somewhat similar, but the contents of the main active components (Cryptotanshinone, Tanshinone I, and Tanshinone IIA) were significantly lower in *S. yunnanensis* radix (Zhang et al., 2019). Similarly, the identification of *S. miltiorrhiza* radix and *S. yunnanensis* radix using NIR, UHPLC, and LC-MS-MS revealed significant differences in the content of lipophilic constituents between the two (Ni et al., 2019). Further clustering analysis of the four components (Salvianolic acid B, Tanshinone I, Tanshinone IIA, and Cryptotanshinone) under the content determination items of *S. miltiorrhiza* radix in the 2020 edition of the “Chinese Pharmacopoeia” showed that *S. miltiorrhiza* radix, *S. yunnanensis* radix, and *S. przewalskii* radix can be clustered into a single branch separately, indicating inconsistency in these four chemical components. Therefore, identifying *S. miltiorrhiza* radix and its adulterants such as *S. yunnanensis* radix, *S. przewalskii* radix, *D. asperoides* radix and the *A. lappa* radix is essential for market regulation and clinical application, and research on the identification of TCM and its adulterants should be strengthened.

Fingerprinting and clustering analysis indicate that *S. miltiorrhiza* radix and its common adulterants are not interchangeable, highlighting the importance of distinguishing *S. miltiorrhiza* radix from its adulterants. Therefore, this study further measured the metabolites of *S. miltiorrhiza* radix and its common adulterants using widely targeted metabolomics technology. Metabolomics is divided into targeted metabolomics, untargeted metabolomics, and a new integrated method—widely targeted metabolomics (Chen et al., 2013). The widely targeted metabolomics technology, based on liquid chromatography-mass spectrometry (LC-MS) and/or gas chromatography-mass spectrometry (GC-MS), combines the precision of targeted analysis with the broadness of untargeted analysis and is widely used in the study of plant secondary metabolism (Yuan et al., 2022). Zhou et al. compared widely targeted metabolomics and untargeted metabolomics of wild *Ophiocordyceps sinensis*, identifying 778 and 1,449 metabolites, respectively. Untargeted metabolomics can rapidly classify samples and obtain more comprehensive metabolic information, while widely targeted metabolomics can focus on specific metabolites related to particular metabolic pathways for quality assessment and metabolite identification (Zhou et al., 2022). Currently, only a few scholars have conducted metabolomic studies on *S. miltiorrhiza* radix and its related species. Zhao et al. used LC/MS technology to analyze the metabolomics of different genotypes and growing environments of *S. miltiorrhiza* radix, finding significant metabolic differences among the same genotype of *S. miltiorrhiza* radix grown in three different locations and three genotypes grown in the same location, and identified 16 and 14 secondary metabolites as specific markers for different growing locations and genotypes of *S. miltiorrhiza* radix, respectively (Zhao Q. et al., 2016). In one study, metabolomic analysis of *S. miltiorrhiza* and *S. prattii* seeds, combined with differences in their antioxidant activities, revealed that the differential compounds included flavonoids and terpenoids (Xia et al., 2023). This study added species outside the genus *Salvia*, *D. asperoides* radix and the *A. lappa* radix, to the analysis of *S. miltiorrhiza* radix and its common adulterants. PCA and PLS-DA analysis revealed chemical differences consistent with previous research, and identified five differential compounds: Hederacoside C, Cardamoni, Emodin, Eriodictyol, and Honokiol, all of which had the highest content in *S. miltiorrhiza* radix. Meanwhile, this study applied widely targeted metabolomics technology to chemically determine *S. miltiorrhiza* radix from ten different regions: Sichuan, Hebei, Shandong, Shanxi, Anhui, Gansu, Henan, Jiangxi, Shaanxi, and Liaoning. PCA and PLS-DA analysis showed that the ten regions could be clearly divided into two groups, with Group a including samples from five origins, namely, Sichuan, Shanxi, Hebei, Henan, and Shaanxi, and Group b including samples from the origins of Anhui, Jiangxi, Liaoning, Shandong, and Gansu. Ipriflavone, Macranthoside B, and Honokiol were identified as differential components, with higher overall contents in *S. miltiorrhiza* radix samples from Group b. These three compounds can serve as chemical markers to distinguish different regions of *S. miltiorrhiza* radix. The differential compounds identified between *S. miltiorrhiza* radix and its adulterants, as well as between different regions of *S. miltiorrhiza* radix, can preliminarily distinguish the origins of *S. miltiorrhiza* radix and differentiate it from its adulterants, providing a theoretical reference for the identification of other TCM materials.

In addition to chemical identification, molecular-level identification techniques are increasingly developing. With advancements in chloroplast genomics research, the chloroplast genomes of various medicinal plants such as *Atractylodes*, *Styrax*, and *Artemisia annua* have been revealed. The entire chloroplast genome evidently contains more informative loci, offering significant advantages in plant genetic development analysis and species identification, serving as a super barcode for plant DNA (Shen et al., 2017; Wang Y. H. et al., 2021; Song et al., 2022). In this study, nine complete chloroplast genes of *S. miltiorrhiza* were obtained. Each chloroplast genome of *S. miltiorrhiza* annotated a total of 132 genes, with lengths ranging from 151,371 to 151,589 bp, similar to other *Salvia* species (Liang et al., 2019). Subsequently, using the nine chloroplast genomes of *S. miltiorrhiza* and 24 other chloroplast genome sequences of *Salvia* species downloaded from NCBI, an NJ phylogenetic tree was constructed. The phylogenetic tree showed that the nine chloroplast genomes of *S. miltiorrhiza* clustered into a single branch, with 100% support, grouped together with *S. yunnanensis* and *S. plectranthoides*, indicating a close phylogenetic relationship between *S. miltiorrhiza* and these two species. The phylogenetic tree indicates that whole chloroplast genome analysis can be used for the identification of *S. miltiorrhiza* and other morphologically similar species within the *Salvia* genus, enabling intrageneric identification of *S. miltiorrhiza*. This study provides a scientific basis for distinguishing *S. miltiorrhiza* from related species, standardizing the cultivation and market circulation of medicinal materials.

Although the chloroplast genome can serve as a super DNA barcode for species identification, it is relatively expensive, and the operation is complex, making large-scale implementation in actual production challenging. At the same time, due to the wide geographical distribution of *S. miltiorrhiza*, the distinction of its origin is also very important, so more convenient and effective method is needed for further identification. Recent studies have found that mutations in the chloroplast genome often occur in hotspot regions (Xu et al., 2017), which vary between species. The *ITS* and *matK* combination sequences are superior barcodes for distinguishing *Astragali* and its adulterants (Guo et al., 2010). Similarly, three candidate DNA barcodes (*trnH-psbA*, *rbcL-α*, *matK*) can distinguish *Sabia parviflora* and its common adulterants to different degrees (Sui et al., 2011). Therefore, highly variable regions of the chloroplast genome can serve as specific DNA barcodes for species identification, useful for distinguishing between and within species. Research on the highly variable regions of *S. miltiorrhiza* has primarily focused on the genus level. Gao et al. compared *S. miltiorrhiza* with *S*. *yangii* and identified ten mutation hotspots (*trnK-rps16, atpH-atpI, psaA-ycf3, ndhC-trnV, ndhF, rpl32-trnL, ndhG-ndhI, rps15-ycf1, ycf1a,* and *ycf1b*) (Gao et al., 2020). Liang et al. compared the chloroplast genomes of four *Salvia* species, identifying candidate segments for distinguishing *Salvia* species: *trnV-ndhC, trnQ-rps16, atpI-atpH, psbA-ycf3, ycf1, rpoC2, ndhF, matK, rpoB, rpoA,* and *accD*. However, there is limited research on highly variable regions within *S. miltiorrhiza* (Liang et al., 2019). In this study, the chloroplast genomes from nine different production area were analyzed using mVISTA and ClustalX software, resulting 6 candidate specific DNA fragments including *rps16-trnQ-UUG, atpF, ycf3, rps4-trnT-UGU, petB-petD,* and *clpP*, which were different from the specific DNA barcodes distinguishing *Salvia* species. Subsequently, we selected the segments *atpF* and *rps4-trnT-UGU*, which are 300–1,300 bp long and have more variable loci, for further study. The aligned lengths of the *atpF* and *rps4-trnT-UGU* sequences were 977 bp and 407 bp, respectively, yielding 34 haplotypes and 22 haplotypes. Combined analysis of the two segments resulted in 62 haplotypes among the 235 samples, with Hap37 being the main haplotype, found in 25 samples from Shanxi Yuncheng, Shanxi Linfen, Henan Nanyang, Henan Luoyang, and Gansu Qingyang, accounting for 11%. Unique haplotypes were found in several locations, such as Henan Luoyang, Shandong Qingdao, Beijing Miyun, and Shanxi Linfen, which can serve as molecular markers for origin identification. Recent reports indicated that DNA could be also extracted from the herbal drugs in market, and the market samples also could be identified the species, haplotypes and production area according the haplotypes analysis in all production areas in China through DNA barcoding. For example, the analyzed results of the commercial *Rhei rhizoma* samples using *matK* gene indicated that 8 genotypes were identified, and the commercial samples are *Rheum palmatume* and mainly from Gansu, Sichuan and Yunan provinces (Man et al., 2022). Our investigation of haplotypes in *S. miltiorrhiza* will be provided a foundation for identifying the genotypes and production areas of herbal drugs in market. Additionally, a phylogenetic tree was constructed using MEGAX software for the 62 haplotypes, *S. yunnanensis*, *S. przewalskii*, and *S. trijuga*. The results showed high similarity among the different haplotypes of *S. miltiorrhiza*, clustering with *S. yunnanensis*, indicating a close phylogenetic relationship between the two species. This study provides a scientific basis for distinguishing *S. miltiorrhiza* from related species, standardizing the cultivation and market circulation of medicinal materials.

Intraspecific genetic diversity is a crucial component of biodiversity, reflecting a species’ evolutionary and environmental adaptability (Wan et al., 2022). Some scholars analyzed the genetic diversity of cultivated *S. miltiorrhiza* from 40 populations collected in China, indicating that cultivated *S. miltiorrhiza* has rich genetic diversity (Feng et al., 2022). This study’s genetic diversity analysis reveals high genetic diversity in multiple locations, with 71.43% of populations having an h_d_ > 0.5, consistent with previous findings, indicating *S. miltiorrhiza*’s strong environmental adaptability. The average genetic distance among the 62 haplotypes of *S. miltiorrhiza* was 0.00226. Hap42 and Hap45 had the greatest genetic distance from Hap24, Hap59, Hap10, Hap32, Hap53, and Hap54, with a distance of 0.00536. The overall genetic distance among haplotypes was relatively small, indicating minimal genetic differentiation among the *S. miltiorrhiza* samples from the 21 populations. These results provide a theoretical foundation for the conservation of *S. miltiorrhiza* germplasm resources, genetic breeding, and origin identification.

## 5 Conclusion

In this study, we selected *S. miltiorrhiza* radix, a commonly used bulk Chinese herbal medicine in the market, as the research object, and synthesized various experimental methods to identify *S. miltiorrhizaits* from different origins and its adulterants. First, it was confirmed by fingerprinting that *S. miltiorrhiza* radix and its adulterants (*S. yunnanensis* radix, *S. przewalskii* radix, *D. asperoides* radix, and *A. lappa* radix) did not have quality consistency, and then five compounds (Hederacoside C, Cardamoni, Emodin, Eriodictyol, and Honokiol) were screened out for the differentiation of *S. miltiorrhiza* radix from *S. yunnanensis* radix, *D. asperoides* radix, and *A. lappa* radix by broad-targeted metabolomics. And three compounds (Ipriflavone, Macranthoside B, Honokiol) were used for the preliminary differentiation of different production areas of *S. miltiorrhiza* radix. Then, by chloroplast whole genome analysis, *S. miltiorrhiza* was distinguished from most of the similar plants within *Salvia* genus such as *S. przewalskii*, *S. trijuga*, and *S. digitaloides*. Finally, 62 haplotypes were formed by joint analysis of two fragments, *atpF* and *rps4-trnT-UGU*, which can be used to discriminate *S. miltiorrhiza* from different geographical origins. Overall, the research achieved discrimination between *S. miltiorrhiza* and adulterants from different families, within the same genus, and among samples from different geographical origins. It provides a scientific basis for the subsequent resource protection, market circulation and origin identification of *S. miltiorrhiza*, and has high application value.

## Data Availability

The data presented in the study are deposited in the National Center for Biotechnology Information (NCBI). The accession numbers of nine chloroplast genomes of *S. miltiorrhiza* are as follows: BankIt2882670 Seq1 PQ540593, BankIt2882670 Seq2 PQ540594, BankIt2882670 Seq3 PQ540595, BankIt2882670 Seq4 PQ540596, BankIt2882670 Seq5 PQ540597, BankIt2882670 Seq6 PQ540598, BankIt2882670 Seq7 PQ540599, BankIt2882670 Seq8 PQ540600, and BankIt2882670 Seq9 PQ540601.
