# Supplementary material for: Identification of Salvia miltiorrhiza germplasm resources based on metabolomics and DNA barcoding
Source: Front Pharmacol. 2025 Jan 7;15:1518906. doi: 10.3389/fphar.2024.1518906 (PMC11753211; doi:10.3389/fphar.2024.1518906)
Supplement: Supplementary file 1 [file Table1.docx]

**Table S1 Information on the origin of *S. miltiorrhiza* radix and its adulterants used in HPLC analysis.**

| Sample number | materials | place of origin | longitude/E | latitude/N | growth type | cultivation period |
| --- | --- | --- | --- | --- | --- | --- |
| S1 | *S. miltiorrhiza* radix | Dezhou, Shandong | 115°45′23″ | 36°24′45″ | cultivated | annual |
| S2 | *S. miltiorrhiza* radix | Jinan, Shandong | 116°21′23″ | 36°02′04″ | cultivated | annual |
| S3 | *S. miltiorrhiza* radix | Xintai, Shandong | 117°44′46″ | 35°54′05″ | cultivated | annual |
| S4 | *S. miltiorrhiza* radix | Nanyang, Henan | 112°21′16″ | 33°26′41″ | cultivated | annual |
| S5 | *S. miltiorrhiza* radix | Luoyang, Henan | 112°28′54″ | 34°41′17″ | cultivated | annual |
| S6 | *S. miltiorrhiza* radix | Shangqiu, Henan | 114°49'33″ | 33°43'05″ | cultivated | annual |
| S7 | *S. miltiorrhiza* radix | Linfen, Shanxi | 111°22′19″ | 35°37′08″ | cultivated | annual |
| S8 | *S. miltiorrhiza* radix | Yuncheng, Shanxi | 110°46′34″ | 34°47′31″ | cultivated | annual |
| S9 | *S. miltiorrhiza* radix | Handan, Hebei | 114°29′02″ | 36°36′55″ | cultivated | annual |
| S10 | *S. miltiorrhiza* radix | Shijiazhuang, Hebei | 114°10′57″ | 38°19′17″ | cultivated | annual |
| S11 | *S. yunnanensis* radix | Kunming, Yunnan | 102°10′15″ | 24°23′47″ | wild | - |
| S12 | *S. yunnanensis* radix | Kunming, Yunnan | 102°10′15″ | 24°23′47″ | wild | - |
| S13 | *S. yunnanensis* radix | Wenshan, Yunnan | 104°15′58″ | 23°21′11″ | wild | - |
| S14 | *S. yunnanensis* radix | Wenshan, Yunnan | 104°15′58″ | 23°21′11″ | wild | - |
| S15 | *S. yunnanensis* radix | Wenshan, Yunnan | 104°15′58″ | 23°21′11″ | wild | - |
| S16 | *S. yunnanensis* radix | Wenshan, Yunnan | 104°15′58″ | 23°21′11″ | wild | - |
| S17 | *S*. *przewalskii* radix | Longnan, Gansu | 104°55′08″ | 33°24′09″ | wild | - |
| S18 | *S*. *przewalskii* radix | Longnan, Gansu | 104°55′08″ | 33°24′09″ | wild | - |
| S19 | *S*. *przewalskii* radix | Longnan, Gansu | 104°55′08″ | 33°24′09″ | wild | - |
| S20 | *S*. *przewalskii* radix | Dingxi, Gansu | 104°57′24″ | 34°55′05″ | wild | - |
| S21 | *S*. *przewalskii* radix | Dingxi, Gansu | 104°57′24″ | 34°55′05″ | wild | - |
| S22 | *S*. *przewalskii* radix | Dingxi, Gansu | 104°57′24″ | 34°55′05″ | wild | - |
| S23 | *D. asperoides* radix | Bozou, Anhui | 115°41′53″ | 33°50′06″ | wild | - |
| S24 | *D. asperoides* radix | Guiyang, Guizhou | 106°07′04″ | 26°11′23″ | wild | - |
| S25 | *D. asperoides* radix | Deyang, Sichuan | 104°30′12″ | 30°26′05″ | wild | - |
| S26 | *D. asperoides* radix | Chengdu, Sichuan | 102°54′04″ | 30°05′02″ | cultivated | triennial |
| S27 | *D. asperoides* radix | Mianyang, Sichuan | 103°45′46″ | 30°42′27″ | cultivated | triennial |
| S28 | *D. asperoides* radix | Mianyang, Sichuan | 103°45′46″ | 30°42′27″ | cultivated | triennial |
| S29 | *A. lappa* radix | Bozou, Anhui | 115°41′53″ | 33°50′06″ | cultivated | triennial |
| S30 | *A. lappa* radix | Shangluo, Shaanxi | 109°56′03″ | 33°52′22″ | wild | - |
| S31 | *A. lappa* radix | Handan, Hebei | 114°29′2″ | 36°36′55″ | cultivated | triennial |
| S32 | *A. lappa* radix | Bozhou, Anhui | 115°41′53″ | 33°50′06″ | cultivated | biennial |
| S33 | *A. lappa* radix | Weifang, Shandong | 119°09′57″ | 36°39′16″ | cultivated | biennial |

**Table S2 Information on the origin of *S. miltiorrhiza* radix and its adulterants used in metabolomics.**

| name | place of origin | longitude/E | latitude/N | growth type | cultivation period |
| --- | --- | --- | --- | --- | --- |
| *S. miltiorrhiza* radix | Chengdu, Sichuan | 102°54′04″ | 30°05′02″ | cultivated | annual |
| *S. miltiorrhiza* radix | Shijiazhuang, Hebei | 114°10′57″ | 38°19′17″ | cultivated | annual |
| *S. miltiorrhiza* radix | Weifang, Shandong | 119°09′57″ | 36°39′16″ | cultivated | annual |
| *S. miltiorrhiza* radix | Yuncheng, Shanxi | 110°46′34″ | 34°47′31″ | cultivated | annual |
| *S. miltiorrhiza* radix | Bozhou, Anhui | 115°41′53″ | 33°50′06″ | cultivated | annual |
| *S. miltiorrhiza* radix | Dingxi, Gansu | 104°57′24″ | 34°55′05″ | cultivated | annual |
| *S. miltiorrhiza* radix | Luoyang, Henan | 112°28′54″ | 34°41′17″ | cultivated | annual |
| *S. miltiorrhiza* radix | Ganzhou, Jiangxi | 115°44′19″ | 26°38′44″ | cultivated | annual |
| *S. miltiorrhiza* radix | Shangluo, Shaanxi | 109°56′03″ | 33°52′22″ | cultivated | annual |
| *S. miltiorrhiza* radix | Chaoyang, Liaoning | 119°52′15″ | 40°55′42″ | cultivated | annual |
| *S. yunnanensis* radix | Wenshan, Yunnan | 104°15′58″ | 23°21′11″ | wild | - |
| *A. lappa* radix | Dezhou, Shandong | 115°45′23″ | 36°24′45″ | cultivated | triennial |
| *A. lappa* radix | Shangluo, Shaanxi | 109°56′03″ | 33°52′22″ | wild | - |
| *A. lappa* radix | Dingxi, Gansu | 104°57′24″ | 34°55′05″ | cultivated | triennial |
| *D. asperoides* radix | Mianyang, Sichuan | 103°45′46″ | 30°42′27″ | cultivated | triennial |
| *D. asperoides* radix | Guiyang, Guizhou | 106°07′04″ | 26°11′23″ | cultivated | triennial |
| *D. asperoides* radix | Ganzhou, Jiangxi | 115°44′19″ | 26°38′44″ | cultivated | triennial |

**Table S3 Information on the origin of *S. miltiorrhiza* radix used in Chloroplast sequencing.**

| sample number | place of origin | longitude/E | latitude/N | growth type | cultivation period |
| --- | --- | --- | --- | --- | --- |
| Sm01 | Bozhou, Anhui | 115°41′53″ | 33°50′06″ | cultivated | annual |
| Sm02 | Changchun, Jilin | 124°18′17″ | 43°05′22″ | cultivated | annual |
| Sm03 | Deyang, Sichuan | 104°30′12″ | 30°26′05″ | cultivated | annual |
| Sm04 | Shijiazhuang, Hebei | 114°10′57″ | 38°19′17″ | cultivated | annual |
| Sm05 | Linyi, Shandong | 118°24′06″ | 35°05′14″ | cultivated | annual |
| Sm06 | Ganzhou, Jiangxi | 115°44′19″ | 26°38′44″ | cultivated | annual |
| Sm07 | Qingyang, Gansu | 107°49′08″ | 35°20′27″ | cultivated | annual |
| Sm08 | Weinan, Shaanxi | 110°13′03″ | 35°04′40″ | cultivated | annual |
| Sm09 | Nanyang, Henan | 112°21′16″ | 33°26′41″ | cultivated | annual |

**Table S4 Information of *S. miltiorrhiza* radix from different origins.**

| place of origin | code | longitude/E | latitude/N | growth type | cultivation period | quantity |
| --- | --- | --- | --- | --- | --- | --- |
| Bozhou, Anhui | AHBZ | 115°41′53″ | 33°50′06″ | cultivated | annual | 2 |
| Changchun, Jilin | JLCC | 125°17′18″ | 43°49′59″ | cultivated | annual | 3 |
| Linyi, Shandong | SDLY | 118°24′06″ | 35°05′14″ | cultivated | annual | 12 |
| Weifang, Shandong | SDWF | 119°09′57″ | 36°39′16″ | cultivated | annual | 3 |
| Qingdao, Shandong | SDQD | 120°23′46″ | 36°18′26″ | cultivated | annual | 12 |
| Shijiazhuang, Hebei | HBSJZ | 114°10′57″ | 38°19′17″ | cultivated | annual | 22 |
| Baoding, Hebei | HBBD | 115°16′08″ | 38°28′28″ | cultivated | annual | 2 |
| Luoyang, Henan | HNLY | 112°28′54″ | 34°41′17″ | cultivated | annual | 20 |
| Nanyang, Henan | HNNY | 112°21′16″ | 33°26′41″ | cultivated | annual | 17 |
| Yuncheng, Shanxi | SXYC | 110°46′34″ | 34°47′31″ | cultivated | annual | 23 |
| Linfen, Shanxi | SXLF | 111°22′19″ | 35°37′08″ | cultivated | annual | 30 |
| Weinan, Shaanxi | SXWN | 110°13′03″ | 35°04′40″ | cultivated | annual | 2 |
| Shangluo, Shaanxi | SXSL | 109°56′03″ | 33°52′22″ | cultivated | annual | 20 |
| Deyang, Sichuan | SCDY | 104°30′12″ | 30°26′05″ | cultivated | annual | 4 |
| Wenshan, Yunnan | YNWS | 104°15′58″ | 23°21′11″ | wild | - | 5 |
| Qingyang, Gansu | GSQY | 107°49′08″ | 35°20′27″ | cultivated | annual | 6 |
| Longnan, Gansu | GSLN | 104°55′08″ | 33°24′09″ | wild | - | 5 |
| Miyun, Beijing | BJMY | 116°39′33″ | 40°13′07″ | cultivated | annual | 10 |
| Huairou, Beijing | BJHR | 116°16′40″ | 40°18'43″ | cultivated | annual | 13 |
| Yangzhou, Jiangsu | JSYZ | 119°41′23″ | 32°18′30″ | wild | - | 9 |
| Ganzhou, Jiangxi | JXGZ | 115°44′19″ | 26°38′44″ | cultivated | annual | 39 |

**Table S5 Chloroplast genome sequence information for evolutionary analysis**

| NO. | species | Serial number |
| --- | --- | --- |
| 1 | *Salvia azurea* | MT156371.1 |
| 2 | *Salvia bulleyana* | MH603954.1 |
| 3 | *Salvia chanroenica* | MW381777.1 |
| 4 | *Salvia glabrescens* | NC067735.1 |
| 5 | *Salvia hispanica* | MN520017.1 |
| 6 | *Salvia karwinskii* | MT156372.1 |
| 7 | *Salvia officinalis* | MZ636550.1 |
| 8 | *Salvia oxyphora* | MT156374.1 |
| 9 | *Salvia plebeia* | MW381779.1 |
| 10 | *Salvia prattii* | MK944407.1 |
| 11 | *Salvia przewalskii* | MH603953.1 |
| 12 | *Salvia roborowskii* | MK944406.1 |
| 13 | *Salvia splendens* | OM617847.1 |
| 14 | *Salvia trijuga* | NC061229.1 |
| 15 | *Salvia umbratica* | MT156375.1 |
| 16 | *Salvia yangii* | MT537168.1 |
| 17 | *Salvia yunnanensis* | MK944405.1 |
| 18 | *Salvia yunnanensis* | MN341012.1 |
| 19 | *Salvia japonica* | KY646163.1 |
| 20 | *Salvia digitaloides* | MN520016.1 |
| 21 | *Salvia digitaloides* | MT156376.1 |
| 22 | *Salvia aerea* | MW752209.1 |
| 23 | *Salvia castanea* | MW752210.1 |
| 24 | *Salvia plectranthoides* | NC053374.1 |

**Table S6 Primer sequences for *S. miltiorrhiza* radix specific fragments**

| gene fragment | primer direction | Primer sequence (5'-3') |
| --- | --- | --- |
| *atpF* | forward | ATGAAAAATGTAACCGATTCTTTCG |
|  | reverse | GACTAATCGGTTATTTCTTTCATCAC |
| *rps4-trnT-UGU* | forward | TATTGAGATTTTTTCCCGGAACGTGG |
|  | reverse | CTATTACTATAATATGGGCCCGCTTAG |

**Table S7 Calculation of similarity of *S. miltiorrhiza* radix fingerprints**

|  | S1 | S2 | S3 | S4 | S5 | S6 | S7 | S8 | S9 | S10 |
| --- | --- | --- | --- | --- | --- | --- | --- | --- | --- | --- |
| S1 | 1.0000 | 0.9980 | 0.9790 | 0.9920 | 0.9960 | 0.9950 | 0.9670 | 0.9960 | 0.9940 | 0.9920 |
| S2 | 0.9980 | 1.0000 | 0.9670 | 0.9840 | 0.9930 | 0.9900 | 0.9510 | 0.9940 | 0.9980 | 0.9980 |
| S3 | 0.9790 | 0.9670 | 1.0000 | 0.9910 | 0.9880 | 0.9880 | 0.9970 | 0.9880 | 0.9650 | 0.9540 |
| S4 | 0.9920 | 0.9840 | 0.9910 | 1.0000 | 0.9910 | 0.9980 | 0.9850 | 0.9910 | 0.9780 | 0.9730 |
| S5 | 0.9960 | 0.9930 | 0.9880 | 0.9910 | 1.0000 | 0.9950 | 0.9750 | 1.0000 | 0.9940 | 0.9880 |
| S6 | 0.9950 | 0.9900 | 0.9880 | 0.9980 | 0.9950 | 1.0000 | 0.9770 | 0.9950 | 0.9860 | 0.9840 |
| S7 | 0.9670 | 0.9510 | 0.9970 | 0.9850 | 0.9750 | 0.9770 | 1.0000 | 0.9760 | 0.9470 | 0.9330 |
| S8 | 0.9960 | 0.9940 | 0.9880 | 0.9910 | 1.0000 | 0.9950 | 0.9760 | 1.0000 | 0.9940 | 0.9880 |
| S9 | 0.9940 | 0.9980 | 0.9650 | 0.9780 | 0.9940 | 0.9860 | 0.9470 | 0.9940 | 1.0000 | 0.9980 |
| S10 | 0.9920 | 0.9980 | 0.9540 | 0.9730 | 0.9880 | 0.9840 | 0.9330 | 0.9880 | 0.9980 | 1.0000 |
| R | 0.9990 | 0.9960 | 0.9860 | 0.9940 | 0.9990 | 0.9970 | 0.9740 | 0.9990 | 0.9940 | 0.9900 |

**Table S8 Similarity calculation of fingerprints of *S. yunnanensis* radix**

|  | S11 | S12 | S13 | S14 | S15 | S16 |
| --- | --- | --- | --- | --- | --- | --- |
| S11 | 1.0000 | 0.9980 | 0.9950 | 0.9940 | 0.9930 | 0.9940 |
| S12 | 0.9980 | 1.0000 | 0.9980 | 0.9970 | 0.9970 | 0.9980 |
| S13 | 0.9950 | 0.9980 | 1.0000 | 0.9990 | 1.0000 | 1.0000 |
| S14 | 0.9940 | 0.9970 | 0.9990 | 1.0000 | 0.9990 | 1.0000 |
| S15 | 0.9930 | 0.9970 | 1.0000 | 0.9990 | 1.0000 | 1.0000 |
| S16 | 0.9940 | 0.9980 | 1.0000 | 1.0000 | 1.0000 | 1.0000 |
| R | 0.9960 | 0.9990 | 1.0000 | 0.9990 | 0.9990 | 1.0000 |

**Table S9 Similarity of fingerprints of *S. przewalskii* radix**

|  | S17 | S18 | S19 | S20 | S21 | S22 |
| --- | --- | --- | --- | --- | --- | --- |
| S17 | 1.0000 | 0.9950 | 0.9880 | 0.9800 | 0.9960 | 0.9970 |
| S18 | 0.9950 | 1.0000 | 0.9830 | 0.9910 | 0.9940 | 0.9970 |
| S19 | 0.9880 | 0.9830 | 1.0000 | 0.9760 | 0.9960 | 0.9920 |
| S20 | 0.9800 | 0.9910 | 0.9760 | 1.0000 | 0.9810 | 0.9860 |
| S21 | 0.9960 | 0.9940 | 0.9960 | 0.9810 | 1.0000 | 0.9980 |
| S22 | 0.9970 | 0.9970 | 0.9920 | 0.9860 | 0.9980 | 1.0000 |
| R | 0.9970 | 0.9980 | 0.9920 | 0.9900 | 0.9980 | 0.9990 |

**Table S10 Calculated similarity of fingerprints of *D. asperoides* radix**

|  | S23 | S24 | S25 | S26 | S27 | S28 |
| --- | --- | --- | --- | --- | --- | --- |
| S23 | 1.0000 | 0.8090 | 0.9090 | 0.9020 | 0.8910 | 0.9050 |
| S24 | 0.8090 | 1.0000 | 0.8340 | 0.9570 | 0.8840 | 0.9310 |
| S25 | 0.9090 | 0.8340 | 1.0000 | 0.9540 | 0.9810 | 0.9670 |
| S26 | 0.9020 | 0.9570 | 0.9540 | 1.0000 | 0.9630 | 0.9920 |
| S27 | 0.8910 | 0.8840 | 0.9810 | 0.9630 | 1.0000 | 0.9770 |
| S28 | 0.9050 | 0.9310 | 0.9670 | 0.9920 | 0.9770 | 1.0000 |
| R | 0.9270 | 0.9290 | 0.9760 | 0.9930 | 0.9840 | 0.9950 |

**Table S11 Similarity of *A. lappa* radix fingerprints**

|  | S29 | S30 | S31 | S32 | S33 |
| --- | --- | --- | --- | --- | --- |
| S29 | 1.0000 | 0.9510 | 0.9470 | 0.9940 | 0.8880 |
| S30 | 0.9510 | 1.0000 | 0.9670 | 0.9750 | 0.8390 |
| S31 | 0.9470 | 0.9670 | 1.0000 | 0.9560 | 0.7820 |
| S32 | 0.9940 | 0.9750 | 0.9560 | 1.0000 | 0.8970 |
| S33 | 0.8880 | 0.8390 | 0.7820 | 0.8970 | 1.0000 |
| R | 0.9860 | 0.9860 | 0.9720 | 0.9960 | 0.8900 |

**Table S12 *S. miltiorrhiza* radix and adulterants metabolite percentage**

| categorization | amount | Percentage/% | categorization | amount | Percentage/% |
| --- | --- | --- | --- | --- | --- |
| flavonoids | 93 | 24.03% | Sugars and Glycosides | 27 | 6.98% |
| terpene | 67 | 17.31% | Nucleotides and their derivatives | 14 | 3.62% |
| alkaloid | 35 | 9.04% | Lipids | 14 | 3.62% |
| organic acid | 34 | 8.79% | Quinones | 12 | 3.10% |
| Amino acids and their derivatives | 30 | 7.75% | Tannins | 2 | 0.52% |
| Phenylpropanoids | 20 | 5.17% | Others | 39 | 10.08% |

**Table S13 Fold change of *S. miltiorrhiza* radix and adulterants**

| BGI_num | Name | SM-AL | SM-DA | SM-SY |
| --- | --- | --- | --- | --- |
| BGI0171 | Benzyl cinnamate | 180.8877 | 805.0123 | 6.0929 |
| BGI0219 | Isomeranzin | 33.5795 | 71.5823 | 3.5835 |
| BGI0225 | Succinic acid | 2.1123 | 4.1606 | 2.0944 |
| BGI0246 | Methyl hexadecanoate | 36.1961 | 147.0878 | 3.6132 |
| BGI0335 | Anemoside B4 | 2.3623 | 91.5219 | 80.3683 |
| BGI0406 | Hederacoside C | 3.2474 | 337.2479 | 68.8612 |
| BGI0487 | Tadalafil | 216.5631 | 801.2426 | 1721.7548 |
| BGI0545 | Cardamoni | 227.3844 | 1029.6560 | 12.5483 |
| BGI0586 | Arctiin | 66.4882 | 90.3989 | 2.3694 |
| BGI0612 | 7-Demethylsuberosin | 99.3048 | 113.7356 | 2.9742 |
| BGI0657 | Daunorubicin hydrochloride | 97.8319 | 109.1057 | 40.5297 |
| BGI0845 | Ipriflavone | 275.2298 | 785.6885 | 3.9797 |
| BGI0846 | Honokiol | 107.9474 | 221.4226 | 10.0121 |
| BGI0875 | Sulindac | 102.0239 | 5.7040 | 16.0831 |
| BGI0909 | Steviolbioside | 108.4790 | 257.5546 | 37.2647 |
| BGI0967 | Cafestol | 28.2766 | 107.2733 | 2.7773 |
| BGI0984 | Aristolochic acid B | 13.8560 | 273.4662 | 2.6804 |
| BGI0999 | Cotoin | 20.6526 | 2.8612 | 3.8429 |
| BGI1002 | Casticin | 133.3384 | 308.7237 | 7.9335 |
| BGI1008 | Isorhamnetin | 35.2788 | 537.2515 | 3.5867 |
| BGI1039 | Formononetin | 62.8217 | 419.0860 | 2.4483 |
| BGI1076 | Iridin | 703.7563 | 1774.9558 | 13.1556 |
| BGI1108 | Imipramine | 245.9031 | 525.8296 | 2.7998 |
| BGI1153 | Alkannin | 64.9943 | 320.2131 | 2.4069 |
| BGI1158 | Emodin | 234.8657 | 1205.5334 | 11.9797 |
| BGI1164 | Pectolinarigenin | 184.6686 | 869.0005 | 2.3993 |
| BGI1166 | Kaempferol | 21.6775 | 30.2428 | 2.2435 |
| BGI1186 | Tetramethylcurcumin | 259.7123 | 760.1744 | 2.1436 |
| BGI1214 | Genistin | 25.3526 | 20.7114 | 2.2149 |
| BGI1243 | Corylin | 593.6078 | 809.0012 | 4.9950 |
| BGI1249 | Stachyose | 83.8641 | 161.2527 | 2.0305 |
| BGI1262 | Irigenin | 57.6201 | 540.0465 | 3.0670 |
| BGI1265 | Galangin | 191.3216 | 1040.2783 | 2.4104 |
| BGI1281 | Eriodictyol | 3.9492 | 56.4834 | 18.1525 |
| BGI1348 | Maltitol | 6.7241 | 21.7439 | 2.4467 |
| BGI1351 | Licochalcone A | 148.3366 | 87.6282 | 2.5354 |
| BGI1422 | Mefenamic acid | 250.2784 | 1031.0311 | 5.3755 |
| BGI1476 | Rubusoside | 105.1248 | 282.4514 | 36.8037 |
| BGI1600 | p-Hydroxy-cinnamic acid | 2.6703 | 16.4682 | 3.0011 |
| BGI1632 | 3-Indoleacetonitrile | 131.1901 | 176.4857 | 108.4101 |
| BGI1656 | 5,7,3'-Trihydroxy-6,4',5'-trimethoxyflavone | 56.3238 | 446.5947 | 3.1094 |
| BGI1683 | 20(R)-Ginsenoside Rg2 | 289.1837 | 5.8681 | 4.9695 |
| BGI1691 | Styraxlignolide F | 97.7109 | 52.6047 | 6.9878 |
| BGI1694 | 5-Methyl-7-methoxyisoflavone | 748.4621 | 172.2799 | 12.0490 |
| BGI1748 | Rosamultin | 35.0702 | 29.3428 | 2.4546 |

**Table S14 VIP scoce of *S. miltiorrhiza* radix and adulterants**

| BGI_num | Name | Comp. 1 | Comp. 2 | Comp. 3 |
| --- | --- | --- | --- | --- |
| BGI0171 | Benzyl cinnamate | 1.3182 | 1.1488 | 1.1644 |
| BGI0191 | Calceolarioside B | 1.4207 | 1.3314 | 1.2104 |
| BGI0206 | Betaine | 1.1428 | 1.1870 | 1.1705 |
| BGI0219 | Isomeranzin | 1.0958 | 0.9379 | 0.9566 |
| BGI0225 | Succinic acid | 1.0691 | 0.8701 | 0.8030 |
| BGI0246 | Methyl hexadecanoate | 1.0845 | 0.9626 | 0.9546 |
| BGI0280 | 3,4,5-Trimethoxybenzoic acid | 1.3691 | 1.4246 | 1.3193 |
| BGI0282 | 3-Hydroxy-2-methyl-4-pyrone | 1.4095 | 1.3449 | 1.2452 |
| BGI0286 | L-Homocitrulline | 1.5925 | 1.5408 | 1.3858 |
| BGI0288 | Scutellarin methyl ester | 1.1220 | 1.0664 | 1.2296 |
| BGI0292 | 7-Ethyl-10-Hydroxy-Camptothecin | 2.0732 | 1.8791 | 1.6875 |
| BGI0303 | Propyl gallate | 2.2129 | 1.9329 | 1.7906 |
| BGI0347 | N-Acetylneuraminic acid | 1.9967 | 1.6469 | 1.4809 |
| BGI0378 | (-)-Syringaresnol-4-O-Β-D-Apiofuranosy | 1.8410 | 1.6201 | 1.5256 |
| BGI0405 | Sweroside | 1.3337 | 1.4349 | 1.3247 |
| BGI0406 | Hederacoside C | 1.9520 | 1.5894 | 1.4428 |
| BGI0426 | 2-Pyrrolidinecarboxylic acid | 1.2034 | 1.2713 | 1.4755 |
| BGI0430 | L (+)-2-Aminobutyric acid | 1.3789 | 1.1475 | 1.0403 |
| BGI0449 | Rutin | 1.1987 | 0.9828 | 0.9325 |
| BGI0463 | UNII:29HK385L3G | 1.8485 | 1.6011 | 1.4387 |
| BGI0478 | Doxepin | 1.2602 | 1.5058 | 1.3650 |
| BGI0480 | 3-O-Acetyl-1α-hydroxytrametenolic acid | 1.3774 | 1.1428 | 1.0908 |
| BGI0484 | (2E)-2-Methyl-2-pentenoic acid | 1.0476 | 1.2015 | 1.1403 |
| BGI0487 | Tadalafil | 1.0875 | 0.8934 | 0.8205 |
| BGI0529 | Alisol B | 1.2474 | 1.1646 | 1.0997 |
| BGI0545 | Cardamoni | 1.7288 | 1.4316 | 1.3823 |
| BGI0580 | Rosmarinic acid | 1.0214 | 1.2524 | 1.4693 |
| BGI0582 | Solamargine | 1.2389 | 1.0473 | 1.0526 |
| BGI0600 | Gentiopicrin | 1.2821 | 1.1876 | 1.2155 |
| BGI0608 | 5-Methylcytidine | 1.8746 | 1.6361 | 1.4869 |
| BGI0615 | Isoquercitrin | 1.4828 | 1.3751 | 1.2446 |
| BGI0647 | Sinensetin | 1.0851 | 1.0759 | 0.9667 |
| BGI0669 | Farrerol | 1.5421 | 1.7552 | 1.5882 |
| BGI0688 | Doxorubicin hydrochloride | 1.3319 | 1.7513 | 2.0753 |
| BGI0702 | Lindenenol | 1.0288 | 0.8386 | 0.8570 |
| BGI0707 | Alisol C 23-acetate | 1.3347 | 1.2996 | 1.1933 |
| BGI0760 | Shanzhiside | 1.8131 | 1.6445 | 1.4830 |
| BGI0777 | Gossypol | 1.3954 | 1.3448 | 1.2266 |
| BGI0793 | Aristolochic acid | 1.8278 | 1.8510 | 1.7356 |
| BGI0794 | Monocrotaline | 1.2986 | 1.0563 | 1.0022 |
| BGI0798 | 5,7-Dihydroxychromone | 1.2815 | 1.0541 | 1.3631 |
| BGI0807 | 4-amino-butyricacimethylester | 1.2160 | 1.2187 | 1.1419 |
| BGI0836 | D-Proline | 1.1356 | 1.2413 | 1.4586 |
| BGI0839 | Maltopentaose | 2.1246 | 1.7294 | 1.5685 |
| BGI0845 | Ipriflavone | 1.0939 | 0.9938 | 1.0783 |
| BGI0846 | Honokiol | 1.6551 | 1.3521 | 1.3616 |
| BGI0864 | Oroxylin A-7-O-β-D-glucuronide | 1.2719 | 1.0579 | 0.9638 |
| BGI0870 | Betamethasone | 1.5636 | 1.2794 | 1.2527 |
| BGI0871 | Nepodin | 1.5386 | 1.2569 | 1.5412 |
| BGI0878 | Caudatin | 2.0837 | 1.9876 | 1.7925 |
| BGI0882 | 4'-O-Glucosylvitexin | 1.8124 | 1.6829 | 1.5239 |
| BGI0891 | Eleutheroside E | 1.1947 | 1.1116 | 1.0020 |
| BGI0916 | Trilobatin | 1.4120 | 1.4402 | 1.3343 |
| BGI0919 | (-)-Gallocatechin gallate | 1.2150 | 1.0573 | 0.9525 |
| BGI0947 | Ferulaldehyde | 1.6042 | 1.3358 | 1.4715 |
| BGI0950 | 4-Guanidinobutyric acid | 1.6195 | 1.5818 | 1.4206 |
| BGI0957 | Oleandrin | 1.0129 | 1.2473 | 1.1555 |
| BGI0959 | Resibufogenin | 3.0021 | 2.6958 | 2.4221 |
| BGI0965 | Jervine | 1.6601 | 1.4249 | 1.2850 |
| BGI0977 | Telocinobufagin | 3.6373 | 3.2371 | 2.9219 |
| BGI0984 | Aristolochic acid B | 1.2201 | 1.1323 | 1.0331 |
| BGI1002 | Casticin | 1.2328 | 1.0451 | 1.0834 |
| BGI1008 | Isorhamnetin | 1.3522 | 1.2171 | 1.1399 |
| BGI1011 | Linarin | 1.3198 | 1.0761 | 0.9714 |
| BGI1018 | Santonin | 1.2966 | 1.1204 | 1.0286 |
| BGI1020 | Cepharanthine | 1.0336 | 1.1661 | 1.0853 |
| BGI1024 | Isoquercitrin | 1.2227 | 1.0105 | 0.9205 |
| BGI1025 | Hyperoside | 1.0718 | 1.0927 | 1.0348 |
| BGI1041 | Isofraxidin | 1.6491 | 1.3491 | 1.5400 |
| BGI1042 | Fraxinol | 1.2638 | 1.0290 | 1.3634 |
| BGI1076 | Iridin | 1.0406 | 0.9050 | 1.1091 |
| BGI1091 | Dexamethasone | 1.6110 | 1.3450 | 1.2611 |
| BGI1108 | Imipramine | 1.0162 | 0.9491 | 0.9975 |
| BGI1134 | Echinocystic acid | 1.6111 | 1.3212 | 1.4965 |
| BGI1135 | Wogonoside | 1.3234 | 1.1602 | 1.0513 |
| BGI1152 | Hematoxylin | 1.9272 | 1.8556 | 1.6664 |
| BGI1158 | Emodin | 1.7214 | 1.4339 | 1.3707 |
| BGI1197 | L-Abrine | 1.2329 | 1.2528 | 1.1308 |
| BGI1200 | Mucic acid | 1.0164 | 0.8268 | 0.8977 |
| BGI1213 | Naringenin 7-O-glucoside (Prunin) | 1.0430 | 0.9298 | 1.4241 |
| BGI1222 | Androsin | 1.2706 | 1.0636 | 1.2267 |
| BGI1243 | Corylin | 1.0254 | 0.9246 | 1.0565 |
| BGI1281 | Eriodictyol | 2.5018 | 2.0442 | 1.8393 |
| BGI1290 | Jujuboside B | 1.2895 | 1.3748 | 1.3092 |
| BGI1295 | Maleamic Acid | 1.1191 | 1.2398 | 1.4581 |
| BGI1299 | Polyphyllin VI | 1.1965 | 0.9732 | 1.2126 |
| BGI1312 | L-Glutamic acid | 2.0664 | 1.7261 | 1.5503 |
| BGI1332 | Folinic acid | 2.0590 | 1.6758 | 1.5773 |
| BGI1354 | Secoxyloganin | 1.5493 | 1.4768 | 1.3644 |
| BGI1385 | L-Tyrosine | 1.2353 | 1.3955 | 1.3104 |
| BGI1422 | Mefenamic acid | 1.1859 | 1.0738 | 1.1113 |
| BGI1427 | Cucurbitacin B | 1.4948 | 1.3328 | 1.1970 |
| BGI1440 | Handelin | 1.3184 | 1.2758 | 1.1606 |
| BGI1459 | N-Methyl-D-aspartic acid | 1.9680 | 1.6367 | 1.4702 |
| BGI1461 | Pinoresinol diglucoside | 1.5801 | 1.3982 | 1.3392 |
| BGI1491 | 6-Demethoxytangeretin | 1.7417 | 1.6909 | 1.5416 |
| BGI1501 | (-)-Syringaresinol di-O-glucoside | 1.2688 | 1.1199 | 1.0089 |
| BGI1504 | Phosphocreatine | 1.4432 | 1.1998 | 1.0823 |
| BGI1537 | D-Glutamic acid | 1.7205 | 1.4004 | 1.2664 |
| BGI1600 | p-Hydroxy-cinnamic acid | 1.3461 | 1.1001 | 0.9881 |
| BGI1606 | Methylophiopogonanone A | 2.4518 | 2.1518 | 1.9379 |
| BGI1623 | Rhynchophylline | 1.9519 | 1.7365 | 1.6270 |
| BGI1691 | Styraxlignolide F | 1.2283 | 0.9990 | 1.1757 |
| BGI1694 | 5-Methyl-7-methoxyisoflavone | 1.2411 | 1.0103 | 1.2524 |
| BGI1705 | Azithromycin | 2.8981 | 2.4135 | 2.1682 |
| BGI1716 | Qingyangshengenin | 1.9083 | 1.9592 | 1.7999 |
| BGI1717 | Rosavin | 1.2300 | 1.1817 | 1.0926 |
| BGI1732 | N-Acetyl-DL-tryptophan | 1.2153 | 1.3578 | 1.2330 |
| BGI1807 | N-Acetyl-DL-serine | 1.7066 | 1.6644 | 1.4963 |
| BGI1808 | Picfeltarraenin IB | 1.8804 | 1.6741 | 1.5087 |
| BGI1815 | Ailanthone | 2.0563 | 1.8331 | 1.7403 |
| BGI1826 | Methyl rosmarinate | 1.0237 | 1.0934 | 1.0742 |

**Table S15 Fold change of *S. miltiorrhiza* radix from ten different origins**

| BGI_num | Name | Fold_Change |
| --- | --- | --- |
| BGI0171 | Benzyl cinnamate | 7.5854 |
| BGI0217 | Lumichrome | 6.9367 |
| BGI0219 | Isomeranzin | 5.0828 |
| BGI0246 | Methyl hexadecanoate | 4.9701 |
| BGI0361 | Aristolactam I | 2.8540 |
| BGI0406 | Hederacoside C | 41.4033 |
| BGI0410 | Deacetylasperulosidic acid | 2.1674 |
| BGI0422 | Macranthoside B | 4.0150 |
| BGI0545 | Cardamoni | 19.4871 |
| BGI0612 | 7-Demethylsuberosin | 7.6993 |
| BGI0618 | Vindoline | 8.9139 |
| BGI0656 | 6-Gingerol | 3.2302 |
| BGI0702 | Lindenenol | 2.0098 |
| BGI0705 | 27-Deoxyactein | 11.7960 |
| BGI0710 | Dehydrodiisoeugenol | 3.9355 |
| BGI0769 | Dehydrocorydaline | 2.0235 |
| BGI0845 | Ipriflavone | 5.7342 |
| BGI0846 | Honokiol | 10.0377 |
| BGI0900 | Ethyl ferulate | 2.1035 |
| BGI0933 | Genkwanin | 2.2853 |
| BGI0941 | Genistein | 2.0486 |
| BGI0967 | Cafestol | 7.3647 |
| BGI0968 | Isoalantolactone | 3.3108 |
| BGI0984 | Aristolochic acid B | 3.0991 |
| BGI0996 | Rheic acid | 2.3083 |
| BGI0999 | Cotoin | 11.0672 |
| BGI1000 | Indirubin | 2.3335 |
| BGI1002 | Casticin | 12.2722 |
| BGI1005 | Oroxylin A | 2.2811 |
| BGI1016 | Acacetin | 2.4789 |
| BGI1023 | Isopimpinellin | 4.9612 |
| BGI1039 | Formononetin | 4.1279 |
| BGI1074 | Luteolin | 4.4130 |
| BGI1077 | Biochanin A | 2.4777 |
| BGI1108 | Imipramine | 5.6657 |
| BGI1153 | Alkannin | 8.0513 |
| BGI1158 | Emodin | 19.8919 |
| BGI1164 | Pectolinarigenin | 5.8930 |
| BGI1166 | Kaempferol | 4.6301 |
| BGI1169 | Tectochrysin | 2.6181 |
| BGI1178 | Emodin-3-methyl ether/Physcion | 2.7937 |
| BGI1186 | Tetramethylcurcumin | 2.6529 |
| BGI1226 | Estrone | 2.2793 |
| BGI1240 | 8-Prenylnaringenin | 3.3906 |
| BGI1243 | Corylin | 9.6260 |
| BGI1262 | Irigenin | 4.3011 |
| BGI1264 | Pinobanksin | 6.9761 |
| BGI1265 | Galangin | 5.6528 |
| BGI1282 | Prunetin | 2.4170 |
| BGI1290 | Jujuboside B | 2.6560 |
| BGI1351 | Licochalcone A | 3.5781 |
| BGI1407 | Tectoridin | 3.9408 |
| BGI1422 | Mefenamic acid | 6.8686 |
| BGI1450 | Wogonin | 2.4787 |
| BGI1623 | Rhynchophylline | 4.5359 |
| BGI1656 | 5,7,3'-Trihydroxy-6,4',5'-trimethoxyflavone | 4.3353 |
| BGI1694 | 5-Methyl-7-methoxyisoflavone | 9.5594 |
| BGI1731 | Dihydrotanshinone I | 2.0816 |
| BGI1806 | Epigallocatechin (-) | 2.8779 |

**Table S16 VIP scoce of *S. miltiorrhiza* radix from ten different origins**

| BGI_num | Name | VIP |
| --- | --- | --- |
| BGI0171 | Benzyl cinnamate | 1.7977 |
| BGI0217 | Lumichrome | 1.6909 |
| BGI0219 | Isomeranzin | 1.7535 |
| BGI0246 | Methyl hexadecanoate | 1.9851 |
| BGI0361 | Aristolactam I | 1.6039 |
| BGI0406 | Hederacoside C | 1.2222 |
| BGI0410 | Deacetylasperulosidic acid | 1.0452 |
| BGI0422 | Macranthoside B | 2.1016 |
| BGI0545 | Cardamoni | 1.8517 |
| BGI0612 | 7-Demethylsuberosin | 1.8313 |
| BGI0618 | Vindoline | 1.2583 |
| BGI0656 | 6-Gingerol | 1.5834 |
| BGI0702 | Lindenenol | 1.6793 |
| BGI0705 | 27-Deoxyactein | 1.1141 |
| BGI0710 | Dehydrodiisoeugenol | 1.6077 |
| BGI0769 | Dehydrocorydaline | 1.2201 |
| BGI0845 | Ipriflavone | 2.1462 |
| BGI0846 | Honokiol | 2.0580 |
| BGI0900 | Ethyl ferulate | 1.3390 |
| BGI0933 | Genkwanin | 1.3656 |
| BGI0941 | Genistein | 1.2799 |
| BGI0967 | Cafestol | 1.7647 |
| BGI0968 | Isoalantolactone | 1.7804 |
| BGI0984 | Aristolochic acid B | 1.7129 |
| BGI0996 | Rheic acid | 1.3812 |
| BGI0999 | Cotoin | 1.8714 |
| BGI1000 | Indirubin | 1.6675 |
| BGI1002 | Casticin | 1.8920 |
| BGI1005 | Oroxylin A | 1.3646 |
| BGI1016 | Acacetin | 1.4316 |
| BGI1023 | Isopimpinellin | 1.5795 |
| BGI1039 | Formononetin | 1.8173 |
| BGI1074 | Luteolin | 1.4593 |
| BGI1077 | Biochanin A | 1.4246 |
| BGI1108 | Imipramine | 1.5899 |
| BGI1153 | Alkannin | 1.6866 |
| BGI1158 | Emodin | 1.8717 |
| BGI1164 | Pectolinarigenin | 1.7135 |
| BGI1166 | Kaempferol | 1.4483 |
| BGI1169 | Tectochrysin | 1.5311 |
| BGI1178 | Emodin-3-methyl ether/Physcion | 1.3835 |
| BGI1186 | Tetramethylcurcumin | 1.1455 |
| BGI1226 | Estrone | 1.2946 |
| BGI1240 | 8-Prenylnaringenin | 1.3561 |
| BGI1243 | Corylin | 1.7928 |
| BGI1262 | Irigenin | 1.7924 |
| BGI1264 | Pinobanksin | 1.6772 |
| BGI1265 | Galangin | 1.6963 |
| BGI1282 | Prunetin | 1.3468 |
| BGI1290 | Jujuboside B | 1.2863 |
| BGI1351 | Licochalcone A | 1.9062 |
| BGI1407 | Tectoridin | 1.2074 |
| BGI1422 | Mefenamic acid | 1.8239 |
| BGI1450 | Wogonin | 1.4307 |
| BGI1623 | Rhynchophylline | 1.2366 |
| BGI1656 | 5,7,3'-Trihydroxy-6,4',5'-trimethoxyflavone | 1.8008 |
| BGI1694 | 5-Methyl-7-methoxyisoflavone | 1.9262 |
| BGI1731 | Dihydrotanshinone I | 1.7573 |
| BGI1806 | Epigallocatechin (-) | 1.5133 |

**Table S17 Characterization of *S. miltiorrhiza* chloroplast genome**

| ID | Total length | LCS | | SSC | | IR | |
| --- | --- | --- | --- | --- | --- | --- | --- |
|  |  | length/bp | GC content/% | length/bp | GC content/% | length/bp | GC content/% |
| Sm01 | 151 589 | 82 841 | 36.10% | 17 580 | 32.00% | 25 584 | 43.11% |
| Sm02 | 151 420 | 82 770 | 36.14% | 17 572 | 31.99% | 25 539 | 43.13% |
| Sm03 | 151 371 | 82 753 | 36.14% | 17 576 | 32.05% | 25 521 | 43.13% |
| Sm04 | 151 584 | 82 826 | 36.13% | 17 578 | 31.98% | 25 590 | 43.10% |
| Sm05 | 151 439 | 82 789 | 36.13% | 17 572 | 31.99% | 25 539 | 43.12% |
| Sm06 | 151 433 | 82 784 | 36.14% | 17 591 | 31.98% | 25 529 | 43.14% |
| Sm07 | 151 572 | 82 821 | 36.11% | 17 583 | 32.00% | 25 584 | 43.11% |
| Sm08 | 151 395 | 82 777 | 36.13% | 17 576 | 32.01% | 25 521 | 43.13% |
| Sm09 | 151 585 | 82 834 | 36.11% | 17 583 | 31.99% | 25 584 | 43.11% |

**Table S18 Chloroplast genome gene annotation table of *S. miltiorrhiza***

| Category | Gene group | Gene name |
| --- | --- | --- |
| Photosynthesis | Subunits of photosystem I | *psaA*, *psaB*, *psaC*, *psaI*, *psaJ* |
|  | Subunits of photosystem II | *psbA*, *psbB*, *psbC*, *psbD*, *psbE*, *psbF*, *psbH*, *psbI*, *psbJ*, *psbK*, *psbL*, *psbM*, *psbN*, *psbT*, *psbZ* |
|  | Subunits of NADH dehydrogenase | *ndhA**, *ndhB**(2), *ndhC*, *ndhD*, *ndhE*, *ndhF*, *ndhG*, *ndhH*, *ndhI*, *ndhJ*, *ndhK* |
|  | Subunits of cytochrome b/f complex | *petA*, *petB**, *petD**, *petG*, *petL*, *petN* |
|  | Subunits of ATP synthase | *atpA*, *atpB*, *atpE*, *atpF**, *atpH*, *atpI* |
|  | Large subunit of rubisco | *rbcL* |
| Self-replication | Proteins of large ribosomal subunit | *rpl14*, *rpl16**, *rpl2**(2), *rpl20*, *rpl22*, *rpl23*(2), *rpl32*, *rpl33*, *rpl36* |
|  | Proteins of small ribosomal subunit | #*rps19*, *rps11*, *rps12***(2), *rps14*, *rps15*, *rps16**, *rps18*, *rps19*, *rps2*, *rps3*, *rps4*, *rps7*(2), *rps8* |
|  | Subunits of RNA polymerase | *rpoA*, *rpoB*, *rpoC1**, *rpoC2* |
|  | Ribosomal RNAs | *rrn16*(2), *rrn23*(2), *rrn4.5*(2), *rrn5*(2)  *trnA-UGC**(2), *trnC-GCA*, *trnD-GUC*, *trnE-UUC*, *trnF-GAA*, *trnG-GCC*, *trnG-UCC**, *trnH-GUG*, *trnI-CAU*(2), *trnI-GAU**(2), *trnK-UUU**, *trnL-CAA*(2), *trnL-UAA**, *trnL-UAG*, *trnM-CAU*, *trnN-GUU*(2),  *trnP-UGG*, *trnQ-UUG*, *trnR-ACG*(2), *trnR-UCU*, *trnS-GCU*, *trnS-GGA*, *trnS-UGA*, *trnT-GGU*, *trnT-UGU*, *trnV-GAC*(2) , *trnV-UAC**, *trnW-CCA*, *trnY-GUA*, *trnfM-CAU* |
|  | Transfer RNAs |  |
| Other genes | Maturase | *matK* |
|  | Protease | *clpP*** |
|  | Envelope membrane protein | *cemA* |
|  | Acetyl-CoA carboxylase | *accD* |
|  | c-type cytochrome synthesis gene  Translation initiation factor | *ccsA*  *infA* |
| Genes of unknown function | Conserved hypothetical chloroplast ORF | #*ycf1*, *ycf1*, *ycf15*(2), *ycf2*(2), *ycf3***, *ycf4* |

Notes: Gene*: Gene with one introns; Gene**: Gene with two introns; #Gene: Pseudo gene; Gene(2): Number of copies of multi-copy genes;

**Table S19 Number of long repetitive sequences in nine *S. miltiorrhiza* chloroplast genomes**

| ID | F | P | R | C | Sum |
| --- | --- | --- | --- | --- | --- |
| Sm01 | 22 | 22 | 0 | 0 | 44 |
| Sm02 | 26 | 28 | 0 | 0 | 54 |
| Sm03 | 22 | 25 | 0 | 0 | 47 |
| Sm04 | 21 | 22 | 0 | 0 | 43 |
| Sm05 | 26 | 29 | 0 | 0 | 55 |
| Sm06 | 26 | 29 | 0 | 0 | 55 |
| Sm07 | 21 | 22 | 0 | 0 | 43 |
| Sm08 | 22 | 25 | 0 | 0 | 47 |
| Sm09 | 21 | 22 | 0 | 0 | 43 |

**Table S20 Comparative sequence analysis table of *S. miltiorrhiza* chloroplast genomes**

| Gene Name | Sequence length/bp | comparison length/bp | Sequence similarity/% |
| --- | --- | --- | --- |
| *ndhF* | 2241 | 2196 | 97.99 |
| *atpF* | 1257 | 1239 | 98.56 |
| *ycf3* | 1947 | 1922 | 98.71 |
| *clpP* | 1920 | 1896 | 98.75 |
| *rpl32* | 171 | 160 | 98.83 |
| *rps7-ndhB* | 334 | 274 | 82.03 |
| *atpA-atpF* | 103 | 85 | 82.52 |
| *trnS-GCU-trnG-UCC* | 696 | 592 | 85.05 |
| *rrn4.5-rrn5* | 255 | 224 | 87.84 |
| *trnG-GCC-trnfM-CAU* | 182 | 161 | 88.46 |
| *petB-petD* | 194 | 175 | 90.2 |
| *psbI-trnS-GCU* | 166 | 150 | 90.36 |
| *rps16-trnQ-UUG* | 1035 | 953 | 92.07 |
| *rps8-rpl14* | 193 | 178 | 92.22 |
| *rps4-trnT-UGU* | 389 | 363 | 93.31 |

**Table S21 *S. miltiorrhiza* *atpF* gene sequence mutation site table (* indicates the same base as the first row, - indicates the alignment gap)**

| haplotype | Variation site/bp | | | | | | | | | | | | | | | | | | | | | | |  |
| --- | --- | --- | --- | --- | --- | --- | --- | --- | --- | --- | --- | --- | --- | --- | --- | --- | --- | --- | --- | --- | --- | --- | --- | --- |
|  | 56 | 251-257 | 280 | 308 | 339-344 | 405 | 411 | 441 | 455 | 404 | 405 | 406 | 407 | 408 | 517 | 536 | 537 | 538 | 605 | 687 | 745 | 809 | 901 | 929 |
| aHap1 | T | - | A | C | - | A | C | - | T | T | T | T | - | - | A | T | T | T | - | G | G | G | A | A |
| aHap2 | * | - | * | * | - | * | * | - | * | * | * | * | T | - | * | * | - | - | - | * | * | * | * | * |
| aHap3 | * | - | * | * | - | * | T | - | G | * | * | - | - | - | * | - | - | - | - | * | * | * | * | * |
| aHap4 | * | - | * | * | - | * | * | A | * | * | * | * | T | - | * | - | - | - | - | * | * | * | * | * |
| aHap5 | * | - | * | * | - | * | T | - | * | * | * | * | T | - | * | * | - | - | - | * | * | * | * | * |
| aHap6 | * | - | * | T | - | * | * | - | * | * | * | * | - | - | * | * | - | - | - | * | * | * | * | C |
| aHap7 | * | - | * | * | - | * | * | - | * | * | * | * | T | - | * | * | * | - | - | * | * | * | * | * |
| aHap8 | G | - | * | * | - | * | T | - | * | * | * | * | - | - | * | * | - | - | - | * | * | * | * | * |
| aHap9 | * | - | C | * | - | * | * | - | * | * | * | * | - | - | * | * | - | - | - | * | * | * | * | * |
| aHap10 | * | - | * | * | - | * | * | A | * | * | * | * | T | - | * | * | * | - | - | * | * | * | * | * |
| aHap11 | * | - | * | * | - | * | T | - | * | * | * | - | - | - | * | * | - | - | - | * | * | * | * | * |
| aHap12 | * | - | * | * | ATATTA | * | T | - | * | * | * | * | T | - | * | * | - | - | - | * | * | * | * | * |
| aHap13 | * | - | * | * | - | * | T | - | * | * | * | * | - | - | * | * | - | - | - | * | * | * | * | * |
| aHap14 | * | - | * | * | - | * | * | - | * | - | - | - | - | - | * | * | * | - | - | * | * | * | * | * |
| aHap15 | * | - | * | * | - | C | * | - | * | * | * | * | - | - | * | * | * | - | - | * | * | * | * | * |
| aHap16 | * | - | * | * | - | * | * | - | * | * | * | * | T | T | * | * | - | - | - | * | * | * | * | * |
| aHap17 | * | - | * | * | - | * | T | - | * | * | * | * | T | - | * | - | - | - | - | * | * | * | * | * |
| aHap18 | * | - | * | * | - | * | * | - | * | - | - | - | - | - | * | * | - | - | - | * | * | * | * | * |
| aHap19 | * | - | * | * | - | * | T | - | * | * | * | * | T | - | * | * | * | - | - | * | * | * | * | * |
| aHap20 | * | - | * | * | TTATTA | * | * | - | * | * | * | * | T | - | * | * | * | - | - | * | * | * | * | * |
| aHap21 | * | - | * | * | - | * | * | - | * | * | * | * | T | T | * | - | - | - | - | * | * | * | * | * |
| aHap22 | * | - | * | * | - | * | T | - | * | * | * | * | - | - | G | * | - | - | T | * | * | * | * | * |
| aHap23 | * | - | * | * | - | * | * | A | * | * | * | * | T | - | * | * | - | - | - | * | * | * | * | * |
| aHap24 | * | - | * | * | - | * | T | - | * | * | * | * | T | T | * | - | - | - | - | * | * | * | * | * |
| aHap25 | * | - | * | * | - | * | * | - | * | * | * | * | - | - | * | * | - | - | - | * | * | * | * | * |
| aHap26 | * | TTATATG | C | * | - | * | * | - | * | * | * | * | - | - | * | * | - | - | - | * | * | * | * | * |
| aHap27 | * | - | * | * | - | * | * | - | * | * | * | * | T | - | * | - | - | - | - | * | * | * | * | * |
| aHap28 | * | - | * | * | - | * | T | - | * | * | * | * | - | - | * | * | - | - | T | * | * | * | * | * |
| aHap29 | * | - | * | * | - | * | * | - | * | * | * | * | T | - | * | * | - | - | - | A | * | A | * | * |
| aHap30 | * | - | * | * | - | * | T | - | * | * | * | * | T | - | * | * | - | - | - | * | A | * | * | * |
| aHap31 | * | - | * | * | - | * | * | - | * | * | * | * | - | - | * | * | - | - | - | * | * | * | C | * |
| aHap32 | * | - | * | * | - | C | * | - | * | * | * | * | - | - | * | * | - | - | - | * | * | * | * | * |
| aHap33 | * | - | * | * | - | * | T | - | * | * | * | * | T | T | * | * | - | - | - | * | * | * | * | * |
| aHap34 | * | - | * | * | - | * | T | - | * | * | * | - | - | - | * | * | * | - | - | * | * | * | * | * |

**Table S22 *S. miltiorrhiza* *rps4-trnT-UGU* gene sequence mutation site table (* indicates the same base as the first row, - indicates the alignment gap)**

| haplotype | Variation site/bp | | | | | | | | | | | | | | | | | | | | | |
| --- | --- | --- | --- | --- | --- | --- | --- | --- | --- | --- | --- | --- | --- | --- | --- | --- | --- | --- | --- | --- | --- | --- |
|  | 53 | 54 | 55 | 56 | 57 | 80 | 101-107 | 150 | 151-153 | 154-155 | 156-161 | 162-165 | 166-167 | 184 | 213 | 221 | 222-231 | 232-240 | 245 | 250 | 277 | 286 |
| rHap1 | T | T | - | - | - | G | - | - | - | - | - | - | - | A | T | T | - | ATTATATAT | G | T | T | C |
| rHap2 | * | * | T | T | T | * | - | - | - | - | - | - | - | * | A | * | - | * | T | G | * | * |
| rHap3 | * | * | T | T | - | * | - | A | - | - | - | TACT | AT | * | A | * | - | * | T | G | * | T |
| rHap4 | * | * | T | T | - | * | - | A | - | - | - | TACT | AT | * | A | * | - | * | T | G | * | * |
| rHap5 | * | * | T | T | - | * | - | - | - | - | - | - | - | * | A | * | - | * | T | G | * | * |
| rHap6 | * | * | T | - | - | * | - | - | - | - | - | - | - | * | * | * | - | * | * | * | * | * |
| rHap7 | - | - | - | - | - | * | - | - | - | - | - | - | - | * | * | * | ATTATCTATT | * | * | * | * | * |
| rHap8 | * | * | T | T | - | * | - | A | CAT | TC | TACTAT | TACT | AT | * | A | * | - | * | T | G | * | T |
| rHap9 | * | * | T | - | - | * | - | A | CAT | - | - | - | AT | * | * | * | - | * | * | * | * | * |
| rHap10 | - | - | - | - | - | * | - | - | - | - | - | - | - | * | * | * | ATTATCTATT | * | * | * | C | * |
| rHap11 | * | - | - | - | - | * | - | - | - | - | - | - | - | * | * | * | - | * | * | * | * | * |
| rHap12 | * | * | - | - | - | * | - | - | - | - | TACTAT | - | - | * | * | * | - | * | * | * | * | * |
| rHap13 | * | * | T | T | - | * | - | - | - | - | - | - | - | C | A | * | - | * | T | G | * | * |
| rHap14 | * | * | T | T | - | T | - | - | - | - | - | - | - | * | * | * | - | * | * | * | * | * |
| rHap15 | * | * | T | - | - | * | - | A | CAT | TC | TACTAT | TACT | AT | * | A | * | - | * | T | G | * | T |
| rHap16 | * | * | - | - | - | * | - | - | - | - | - | - | - | * | A | * | - | * | T | * | * | * |
| rHap17 | - | - | - | - | - | * | - | - | - | - | - | - | - | * | * | - | - | - | * | * | * | * |
| rHap18 | * | * | T | T | - | * | - | - | - | - | TACTAT | - | - | * | * | * | - | * | * | * | * | * |
| rHap19 | * | * | T | - | - | * | ACTAAAT | - | - | - | - | - | - | * | A | * | - | * | T | G | * | * |
| rHap20 | * | * | - | - | - | * | - | - | - | - | - | - | - | * | A | * | - | * | T | * | * | * |
| rHap21 | * | - | - | - | - | * | - | - | - | - | TACTAT | - | - | * | * | * | - | * | * | G | * | * |
| rHap22 | * | * | - | - | - | * | - | - | - | - | - | - | - | * | * | * | - | * | * | * | * | * |

**Table S23 Haplotype distribution of *S. miltiorrhiza* samples from different origins**

| Collection site code | haplotype distribution |
| --- | --- |
| AHBZ | Hap32(1), Hap3^*^(1) |
| JLCC | Hap21(3) |
| SDLY | Hap1(2), Hap12(1), Hap22(1), Hap33(1), Hap36^*^(1), Hap18(1), Hap29^*^(1), Hap52^*^(1), Hap21(1) |
| SDWF | Hap10(3) |
| SDQD | Hap10(1), Hap19(2), Hap53(1), Hap47(1), Hap59^*^(1), Hap15(1), Hap12(2), Hap14^*^(1), Hap40(1), Hap38(1) |
| HBSJZ | Hap9(3), Hap18(3), Hap2(1), Hap20^*^(1), Hap35^*^(1), Hap16^*^(13) |
| HBBD | Hap26^*^(1） |
| HNLY | Hap42(12), Hap43(1), Hap45^*^(1), Hap18(1), Hap25(1), Hap37(1), Hap41(1) |
| HNNY | Hap8^*^(2), Hap27^*^(3), Hap1(3), Hap37(1), Hap43(1), |
|  | Hap61^*^(1), Hap62^*^(1) |
| SXYC | Hap31^*^(11), Hap1(1), Hap2(4), Hap7^*^(1), Hap37(1), Hap38(3), Hap56^*^(2) |
| SXLF | Hap10(1), Hap12(2), Hap38(1), Hap9(2), Hap32(3), Hap39^*^(1), Hap60^*^(1), Hap37(19) |
| SXWN | Hap10(1), Hap12(1) |
| SXSL | Hap18(1), Hap25(1), Hap33(1), Hap41 (1), Hap44^*^(1), Hap11^*^(1), Hap43(8), Hap9(1), Hap34^*^(1), Hap42(1), Hap51^*^(1) |
| SCDY | Hap1(1), Hap18(1) |
| YNWS | Hap54^*^(1), Hap2(1), Hap12(1) , Hap22(1） |
| GSQY | Hap17^*^(1), Hap37(3), Hap2(1), Hap4^*^(1) |
| GSLN | Hap10(1), Hap19(1), Hap47(1) |
| BJMY | Hap24^*^(7), Hap28^*^(3) |
| BJHR | Hap2(4), Hap13^*^(2), Hap18(2), Hap33(1), Hap46(2), Hap57^*^(1) |
| JSYZ | Hap53(6) |
| JXGZ | Hap1(1), Hap5^*^(1), Hap6^*^(1), Hap10(5), Hap12(5), Hap15(1), Hap18(3), Hap19(2), Hap22(2), Hap23^*^(1), Hap30^*^(1), Hap40^*^(1), Hap46(3), Hap48^*^(1), Hap49^*^(2), Hap50^*^(1), Hap53(1), Hap55^*^(3), Hap58^*^(1) |

Note: * represents origin-specific haplotypes; () is the number of haplotypes.

**Table S24 Genetic diversity parameters of S. *miltiorrhiza* based on cpDNA**

| location | S | h | h_d_ | Pi×10^-3^ |
| --- | --- | --- | --- | --- |
| AHBZ | 5.0000 | 2.0000 | 1.0000 | 3.7700 |
| JLCC | 0.0000 | 1.0000 | 0.0000 | 0.0000 |
| SDLY | 8.0000 | 5.0000 | 0.8060 | 2.4800 |
| SDWF | 0.0000 | 1.0000 | 0.0000 | 0.0000 |
| SDQD | 9.0000 | 7.0000 | 0.9330 | 2.2000 |
| HBSJZ | 4.0000 | 2.0000 | 0.5330 | 1.6100 |
| HBBD | 0.0000 | 1.0000 | 0.0000 | 0.0000 |
| HNLY | 8.0000 | 6.0000 | 0.9520 | 2.3700 |
| HNNY | 3.0000 | 2.0000 | 0.2860 | 0.6500 |
| SXYC | 2.0000 | 2.0000 | 0.2860 | 0.4300 |
| SXLF | 5.0000 | 5.0000 | 0.8930 | 1.8900 |
| SXWN | 1.0000 | 2.0000 | 1.0000 | 0.7500 |
| SXSL | 9.0000 | 5.0000 | 0.7090 | 2.0100 |
| SDZJ | 4.0000 | 2.0000 | 1.0000 | 3.0100 |
| YNWS | 5.0000 | 3.0000 | 0.8330 | 1.8800 |
| GSQY | 4.0000 | 3.0000 | 0.8330 | 1.5100 |
| GSLN | 4.0000 | 3.0000 | 1.0000 | 2.0100 |
| BJMY | 6.0000 | 2.0000 | 1.0000 | 4.5200 |
| BJHR | 6.0000 | 5.0000 | 0.9330 | 2.2800 |
| JSYZ | 0.0000 | 1.0000 | 0.0000 | 0.0000 |
| JXGZ | 11.0000 | 10.0000 | 0.9120 | 2.3600 |
