# Supplementary figures and images for: Identification of Salvia miltiorrhiza germplasm resources based on metabolomics and DNA barcoding
Source: Front Pharmacol. 2025 Jan 7;15:1518906. doi: 10.3389/fphar.2024.1518906 (PMC11753211; doi:10.3389/fphar.2024.1518906)

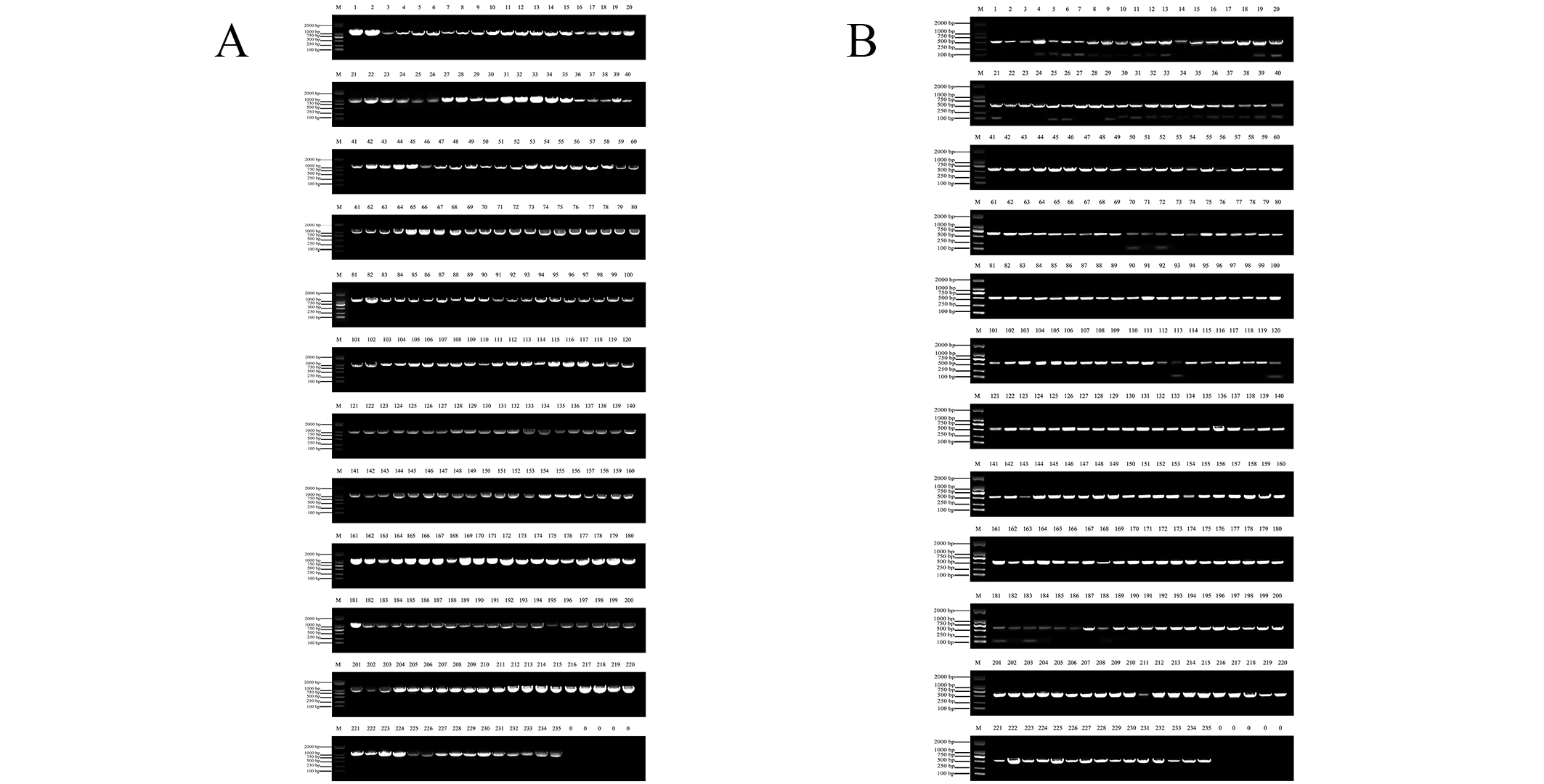

Supplement: Supplementary file 2 [file Image1.tif]
